# Current evidence on the adoption of indicator condition guided testing for HIV in western countries: A systematic review and meta-analysis

**DOI:** 10.1016/j.eclinm.2021.100877

**Published:** 2021-05-08

**Authors:** S.J. Bogers, S.H. Hulstein, M.F. Schim van der Loeff, G.J. de Bree, P. Reiss, J.E.A.M van Bergen, S.E. Geerlings, HIV Transmission Elimination AMsterdam (H-TEAM) Consortium

**Affiliations:** a Department of Internal Medicine, Division of Infectious Diseases, Amsterdam University Medical Centers, location Academic Medical Center, University of Amsterdam, room D3-226, Meibergdreef 9, Amsterdam 1105, Netherlands; b Department of Infectious Diseases, Public Health Service of Amsterdam, Amsterdam, Netherlands; c Amsterdam Institute for Global Health and Development, Amsterdam, Netherlands; d Department of Global Health, Amsterdam University Medical Centers, location Academic Medical Center, University of Amsterdam, Amsterdam, Netherlands; e HIV Monitoring Foundation, Amsterdam, Netherlands; f Department of General Practice, Amsterdam University Medical Centers, location Academic Medical Center, Netherlands; g STI AIDS Netherlands, Amsterdam, Netherlands

## Abstract

**Background:**

Indicator condition (IC) guided testing for HIV is an effective way to identify undiagnosed people living with HIV, but studies suggest its implementation is lacking. This systematic review provides an overview of the adoption of IC-guided testing in Western countries.

**Methods:**

Seven ICs were selected: tuberculosis (TB), malignant lymphoma, hepatitis B, hepatitis C, cervical/vulvar carcinoma/intraepithelial neoplasia grade 2+ (CC/CIN2+, VC/VIN2+), and peripheral neuropathy (PN). Embase and Ovid MEDLINE were searched up to November 20th, 2020. Publications of all types, using data from ≥2009, reporting on HIV test ratios in patients ≥18 years in all settings in Western countries were eligible. HIV test ratios and positivity were reported per IC. A random effects-model for proportions was used to calculate estimated proportions (ES) with 95% CIs. This study was registered at PROSPERO, registration number CRD42020160243.

**Findings:**

Fifty-seven references, including 23 full-text articles and 34 other publications were included. Most (28/57) reported on HIV testing in TB. No reports on HIV testing in VC/VIN2+ or PN patients were eligible for inclusion. Large variation in HIV test ratios was observed between and within ICs, resulting from different testing approaches. Highest HIV test ratios (pooled ratio: 0·72, 95%CI 0·63–0·80) and positivity (0·05, 95% CI 0·03–0·06) were observed among TB patients, and lowest among CC/CIN2+ patients (pooled ES test ratio: 0·12, 95%CI 0·01–0·31, positivity: 0·00, 95%CI 0·00–0·00).

**Interpretation:**

IC-guided HIV testing is insufficiently implemented in Western countries. The large variation in test ratios provides insight into priority areas for implementing routine IC-guided HIV testing in the future.

**Funding:**

HIV Transmission Elimination in Amsterdam (H-TEAM) consortium and Aidsfonds (grant number P-42,702).

**Research in context**

**Evidence before this study**

Identifying and treating people living with HIV is key to control the HIV epidemic. Opportunities to identify undiagnosed HIV through indicator condition guided testing are being missed in various healthcare settings. We have found no systematic review on the extent to which indicator condition guided testing has been adopted in Western countries

We searched Embase and Ovid MEDLINE for evidence published up to November 20th, 2020 for evidence on the extent to which indicator condition guided testing for HIV is implemented in all healthcare settings in the Western world. All publication types, including full-text peer reviewed articles, as well as abstracts, short communications, and correspondence reporting on HIV testing in a selection of seven indicator conditions in or after 2009 in all healthcare settings in Western Countries were eligible for inclusion. No language restrictions were applied. The search included terms for HIV testing, the selected indicator conditions, and the term ‘indicator condition’.

**Added value of this study**

This systematic review revealed that indicator condition guided testing for HIV is unevenly and insufficiently adopted across indicator conditions and healthcare settings in Western countries. We found that even in AIDS defining conditions, such as tuberculosis or cervical cancer, HIV testing strategies need to be further improved. Additionally, for some indicator conditions such as peripheral neuropathy, no evidence on HIV testing was identified, suggesting that in some conditions, this testing strategy might be lacking even more. Overall, this review revealed that adopting these strategies is an effective way to identify undiagnosed HIV, as in most conditions positivity percentages exceeded the established cost-effectivity threshold of 0·1%.

**Implications of all the available evidence**

Indicator condition guided testing for HIV remains insufficiently practiced, and results from settings where this strategy is best implemented reveal opportunities for improvement. Lessons on effective implementation can be learned from these settings, such as the high HIV test ratio observed when opt-out testing strategies are used. Adopting these strategies could lead to improved indicator condition guided HIV testing strategies across healthcare settings in Western countries.

Alt-text: Unlabelled box

## 1 Introduction

In our global efforts to complete the ‘last mile’ towards ending the HIV epidemic, timely diagnosis remains an important challenge. In the European Union/European Economic Area (EU/EEA), an estimated 14% of people living with HIV (PLHIV) is unaware of their diagnosis and late diagnosis (CD4 count <350 cells/mm^3^) is reported in almost half of all new cases. [1] These figures are of particular concern, as late presentation is associated with higher morbidity, mortality, and onward transmission of HIV. [2,3]

In the last decade, growing evidence on the potential role of indicator condition guided testing for HIV to improve timely testing has emerged. Indicator conditions (ICs) are defined as conditions that are either (1) AIDS-defining, (2) associated with an undiagnosed HIV prevalence of >0·1%, the cut-off for cost-effective screening for HIV, [4,5] or (3) conditions where failure to identify an HIV infection may have significant adverse implications for the patient. [6] In 2007 the World Health Organization recommended provider-initiated HIV testing in conditions that could indicate HIV infection, [7] and in 2014 the HIV in Europe-initiative published a guidance on IC-guided HIV testing in adults, [6] based on the HIV Indicator Diseases across Europe Study (HIDES) and subsequent HIDES II study, that were performed in Europe. [8,9]

In recent years numerous studies have shown that IC-guided HIV testing is an effective approach to identify undiagnosed PLHIV. [10], [11], [12], [13], [14], [15], [16], [17] Additionally, IC-guided testing has the advantage of bypassing barriers on both the patient and provider level, such as discussing sexual behavior and risk factors for HIV. [8] As a consequence, HIV guidelines recommend IC-guided testing as one of the strategies to reduce the proportion undiagnosed PLHIV. However, recent studies on implementation of IC-guided testing consistently show missed opportunities for earlier HIV diagnosis due to lack of adherence to- or absence of- local protocols on IC-guided testing, but no overview of the adoption of IC-guided testing has been reported. [18], [19], [20], [21], [22], [23], [24], [25]

The main objective of this systematic review was to assess the proportion of patients presenting with indicator conditions that are tested for HIV (i.e. the HIV test ratio). The secondary objective was to assess the outcomes of this testing strategy (i.e. the percentage positive).

## 2 Methods

### 2.1 Protocol and guidelines

The protocol for this review was published at PROSPERO (**supplementary appendix 1**), and reported in accordance with the Preferred Reporting Items for Systematic Reviews and Meta-Analyses (PRISMA) statement (**supplementary appendix 2**).

### 2.2 Review topics

Seven ICs from various medical specialties were selected for inclusion: tuberculosis (TB), cervical cancer (CC) or cervical intraepithelial neoplasia (CIN) grade 2+, vulvar cancer (VC) or vulvar intraepithelial neoplasia (VIN) grade 2+, malignant lymphoma, hepatitis B (HBV), hepatitis C (HCV), and peripheral neuropathy (PN). These ICs were selected as they are diagnosed and managed by various medical specialties (i.e. pulmonology, gynecology, hematology, gastroenterology/hepathology and neurology), ensuring a wide scope of the extent to which IC-guided testing is adopted, and they can all be objectively diagnosed using diagnostic tests.

### 2.3 Search strategy

With assistance of a clinical librarian, Ovid MEDLINE and Embase were searched for studies published up to November 20th, 2020. The search contained terms for HIV testing, the selected ICs, and the term ‘indicator condition’ (**supplementary appendix 3**). No language or date restrictions were applied. Additionally, all articles referring to the HIDES studies, [8,9] and abstracts identified in Embase were included for screening.

### 2.4 Selection criteria

Studies reporting HIV test ratios among patients ≥18 years (directly available or through calculation with presented data), all settings (e.g. primary care (PC), hospital care, registry surveillance), and all publication types (e.g. research article, abstract, correspondence) were eligible for inclusion. Only studies performed in Western countries (Western Europe, USA, Canada, Australia, New Zealand, and Japan) were included, as HIV epidemiology and the standard of healthcare are comparable in these countries. No language restrictions were applied. Studies among persons known HIV positive, with unconfirmed disease (e.g. suspected TB), or conditions not meeting the IC definition (e.g. latent TB infection), and studies with a sample size <10 per subgroup per IC were excluded. Studies with data on HIV testing before 2009 only were excluded, as IC-guided testing was globally implemented around 2009.

### 2.5 Selection process

Search results were exported through an EndNote database (version 19·1, Thomson Reuters, Philadelphia, USA) and duplicates were removed. All titles and abstracts were screened for inclusion by SJB, and 10% were independently screened by SHH. A maximum of 2·5% discrepancy was allowed for. Differences were resolved through discussion, and, if needed, SEG was consulted as a third reviewer to resolve differences of opinion. If after discussion the discrepancy remained >2·5%, all titles and abstracts would be screened by SHH. Subsequently, the full text of all selected references was assessed for eligibility by both reviewers. Differences were again resolved through discussion. For all eligible abstracts, subsequent full-text publications were searched for.

### 2.6 Data extraction

For data extraction, a form in Microsoft Excel (Version 2016, Microsoft Corporation, USA) was used. The form was piloted in the first 10% of eligible studies, and adjusted accordingly. Data extraction was independently performed by SJB and SHH and discrepancies were resolved through discussion, with consultation of SEG as a third reviewer, if needed. Type of publication (i.e. full-text peer-reviewed article or ‘other publication types’, including abstracts, short communications, and correspondence), first author, year, title, setting, aim, recruitment site, definition of the IC and of being HIV tested, inclusion and exclusion criteria, number of subjects, number tested for HIV, and data on the percentage positive were extracted, **supplementary appendix 4.** When HIV test ratios were presented separately by sex or time periods (e.g. before and after intervention), they were extracted separately. Missing data were requested from authors if needed.

### 2.7 Quality assessment

Risk of bias assessment per included full-text study was performed independently by SJB and SHH using an adaptation of the Joanna Briggs checklist for prevalence studies, with consultation of SEG as a third reviewer, if needed. [26] The item on statistical analysis was dropped as it was deemed not relevant, and an item on objective measurement of being HIV tested was added (**supplementary appendix 5**). Risk of bias was scored out of 10. Discrepancies were resolved by discussion. No risk of bias was assessed for the other publication types (including abstracts, short communications, and correspondence, as insufficient information was available in these publications.

### 2.8 Statistical analysis

HIV test ratios, percentage positive, and quality assessments per reference were reported by IC. Summary statistics across studies were reported as medians with interquartile ranges (IQR). Test ratios and positivity were pooled by IC, regardless of publication type and assessed risk of bias. A random effects-model for proportions by Nyaga et al. was used, [27] as considerable heterogeneity between studies was expected due to the broad inclusion criteria. No limit for heterogeneity as expressed by the I^2^ statistic was used. Results were reported as estimated proportions (ES) and 95% confidence intervals (CI) and displayed as forest plots. In sensitivity analyses, pooling of test ratio was performed using only low risk of bias full-text articles, and stratified by sex. A risk of bias score of ≥7/10 was chosen as cut-off for low risk by the researchers. Additionally, meta-regression analyses of the HIV test ratio per study by date of data collection (as a continuous variable) were performed to assess whether HIV test ratio varied by time, overall and by IC. For date of data collection, the midpoint of reported periods were taken. Permuted tests with an iteration of 1000 were used to confirm the findings. Analyses were performed using STATA 15 (StataCorp LLC, College Station, USA).

### 2.9 Role of funding sources

The funders of this study had no role in the study's design, conduct, analysis and interpretation of results, the writing of the report, or the decision to publish.

## 3 Results

A total of 3405 records, including 992 abstracts and 62 records referencing the HIDES studies were identified through the search. Eighty-three were excluded because they were duplicates and 3219 based on title/abstract. Less than 2·5% discrepancy was found between the two screening authors during independent screening (5/341, 1·5%), which was resolved through discussion. Of the remaining 103 references, 46 were excluded based on full-text screening.

**Fig. 1:**
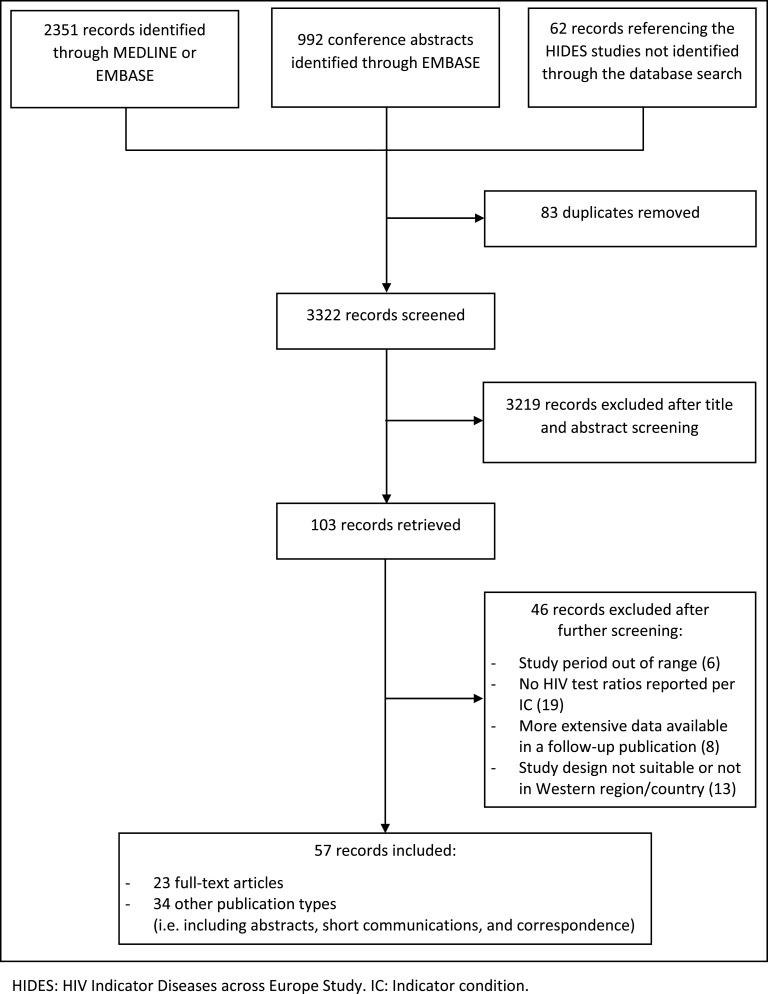
Search results and inclusions. *HIDES: HIV Indicator Diseases across Europe Study. IC: Indicator condition.*

Of the 57 included references reporting on one or more IC, 23 were full-text articles and 34 were other publication types including abstracts, short communications, and correspondence **(**Fig. 1**).** Three of the 57 included citations reported on four or five of selected ICs, two reported on three ICs, ten reported on two ICs and 42 reported on one. Most included records (28/57) reported on HIV testing in TB patients **(**Table 1**)**. No records on HIV testing in VC/VIN2+ patients or PN patients were eligible for inclusion. Most records were from the UK (24), followed by the USA (14) and Canada (5). Twenty-four records had included data from prior to 2009. There was considerable variation between records in how ‘tested for HIV’ was defined; 37% of studies (21) had defined a timeframe for being tested, using varying timeframes. Forty percent (23) described how HIV testing was defined, but did not define a timeframe, and 23% (13) described no definition of ‘HIV tested’, despite HIV test ratios being reported.

**Table 1:** Included records per indicator condition and publication type*.

	Number of included full-text publications	Number of included other publication types **	Total included citations
Tuberculosis	16	12	28
Hepatitis B	3	9	12
Hepatitis C	5	13	18
Hepatitis B or C	2	1	3
Cervical carcinoma or CIN2+	6	3	9
Vulvar carcinoma or VIN2+	0	0	0
Malignant lymphoma	4	7	11
Peripheral neuropathy	0	0	0
**Total**	**23**	**34**	**57**

* Numbers add up more than the total number of included citations, as some reported on more than one indicator condition.

** (i.e. abstracts, short communications, and correspondence).

CIN: cervical intraepithelial neoplasia; VIN: vulvar intraepithelial neoplasia.

### 3.1 Tuberculosis

Of 16 included full-text articles on TB, eight were performed in a hospital/TB clinic setting, seven in the setting of a TB registry database, and one in the PC setting. Median number of study subjects was 603 (IQR 340–1355). HIV test ratios ranged from 44% to 95% in the hospital/registry setting, and was 8% in the PC setting. Median positivity percentage was 4·9% (IQR 4·4%−5·8%). Risk of bias was low; 77% of full-text references (13/16) had a low risk assessment (7/10 or higher). Across the 12 included other publication types, median number of subjects was 219 (IQR 28–463) and median HIV test ratio was 72% (IQR 56%−92%), Table 2.

**Table 2:** Study characteristics, summary of findings and risk of bias by indicator condition.

Tuberculosis								
Full-text articles								
Reference (year)	Design and setting	Included study period	Population and exclusion criteria	IC definition	HIV tested definition	HIV test ratio (%)**	Positivity ratio (%)**	Risk of bias score*
Anderson (2013) [33]	Retrospective cohort study in UK TB clinics – before cohort implementation	July 2009 - June 2010	All TB cases of all ages from 5 London clinics were included	Patients notified as having TB disease	Uptake of HIV testing	Before: 510/557 (91·6%)	NA	7/10
	Retrospective cohort study in UK TB clinics – after cohort implementation	July 2010 - December 2011				After: 687/752 (91·4%)		
Augusti (2016) [19]	Cross-sectional cohort in primary care, Spain	January 2010 - August 2012	Patients aged 16–65 years were included; known HIV positive patients excluded	Using either their ICD-10 codes or a positive laboratory result	HIV test within 4 months of diagnosis date	Men: 112/1287 (8·7%)	Men: 0/112 (0%)	9/10
						Women: 63/840 (7·5%)	Women: 1/63 (1·6%)	
Basham (2018) [28]	Audit of a Canadian provincial tuberculosis program	2008 - 2010	All active TB cases of all ages in the TB registry	Active TB	HIV test recorded in TB registry database	250/428 (58·4%)	12/250 (4·8%)	9/10
Basham (2019) [39]	Audit of First Nations tuberculosis program in Canada	2008 - 2012	First Nations of all ages recorded in the TB registry	Recorded TB in registry	HIV test recorded in TB registry database	95/149 (63·8%)	NA	8/10
Clark (2013) [40]	Retrospective cohort to assess HIV testing in TB surveillance database in US	2008 - 2010	Living patients with TB of all ages	Reported TB cases surviving with TB	Known (positive or negative) or unknown (refused testing/not offered testing) HIV status	208/273 (76·2%)	12/208 (5·8%)	7/10
Clerk (2013) [41]	Cross-sectional study on HIV testing in TB patients in the UK	NA	TB cases of all ages were included	Confirmed active TB cases	NA	27/31 (87·1%)	NA	6/10
Gardner (2012) [42]	Retrospective cohort after implementation of opt-out HIV testing in US TB clinic	June 2010 - June 2011	Excluded: Patients <14 years, known HIV positive, no chart available, diagnosed prior to study period	New TB cases presenting at the clinic	Tested for HIV in the clinic after presentation	458/939 (48·8%)	1/458 (0·2%)	9/10
Gupta (2011) [35]	First audit of IC-guided testing in UK general hospital	First audit: August 2008 - July 2009	Patients of all ages testing positive for TB	Patients tested positive for tuberculosis	HIV testing was double checked using the electronic pathology records system and a separate database of HIV testing	First audit: 19/25 (76·0%)	NA	7/10
	*Re*-audit of IC-guided testing in UK general hospital	*Re*-audit: August 2009 - June 2010				*Re*-audit: 12/29 (41·4%)		
Long (2014) [29]	Retrospective cohort of tuberculosis patients in Canada	2003–2012	Patients of all ages in the TB registry	Persons meeting the Canadian case definition for TB	Already known or newly diagnosed with HIV	1317/1453 (90·6%)	74/1317 (5·6%)	8/10
Post (2015) [43]	Retrospective cohort among patients with tuberculosis in Australia	2009	Patients of all ages with TB	Microbiologically confirmed TB and patients that were treated for TB without microbiological confirmation	HIV status was categorised as known or unknown (not tested or declined testing)	2009: 56/80 (70·0%)	2009: 3/56 (5·4%)	6/10
		2010				2010: 79/100 (79·0%)	2010: 5/79 (6·3%)	
		2011				2011: 72/98 (73·5%)	2011: 4/72 (5·6%)	
		2012				2012: 56/73 (76·7%)	2012: 1/56 (1·8%)	
		2013				2013: 61/70 (87·1%)	2013: 3/61 (4·9%)	
Raben (2015) [9]	Retrospective cohort on HIV testing in ICs in Europe	May 2013	Patients in participating centres, >18 and <65 years of age, not known HIV positive, presenting within the previous year/last 100+ patients	Patients with tuberculosis	Participating centres reviewed retrospectively how many patients presenting with the IC were tested for HIV	1041/1401 (74·3%)	46/1041 (4·4%)	2/10
Ribeiro (2018) [44]	Retrospective cohort study on HIV screening of TB patients in Portugal	2008 - 2014	Notified TB cases of all ages in the Portuguese Tuberculosis Surveillance System	Notified TB	NA	Men: 10,629/12,115 (87·7%)	Men: NA	7/10
						Women: 5414/6330 (85·5%)	Women: NA	
Rivest (2014) [45]	Retrospective cohort on HIV-TB co-infection and predictors of HIV screening among incident TB cases in Canada	2004 - 2009	Incident TB cases of all ages reported to the TB reporting database	Cases confirmed by culture or diagnosed on the basis of clinical and radiological signs	HIV testing done from one month before to six months after date of TB diagnosis	Men: 226/422 (53·6%)	Men: 26/226 (11·5%)	8/10
						Women: 169/356 (47·5%)	Women: 7/169 (4·1%)	
Roy (2013) [36]	Cluster randomised controlled trial on the impact of implementing universal HIV testing in TB patients in the UK	September 2009 - March 2010	Patients of all ages in centres using a selective HIV testing policy, not known HIV infected.	All patients seen and diagnosed with TB in participating centres	The date the HIV test was conducted was recorded and these patients were classified as having ‘‘accepted’’ the test	Women (selective testing): 269/417 (64·5%)	NA	7/10
						Men (selective testing): 376/544 (69·1%)		
			Patients of all ages in centres using a universal HIV testing policy, not known HIV infected.			Women (universal testing): 111/149 (74·5%%)		
						Men (universal testing): 152/198 (76·8%)		
Sewell (2014) [46]	Retrospective cohort in a UK TB clinic	January 2009 - July 2012	TB patients of all ages at a TB medical outpatient service	Clinical or laboratory TB diagnosis	Tested < 3 months of attending the clinic or starting TB treatment	389/410 (94·9%)	27/389 (6·9%)	9/10
William (2011) [47]	Audit on HIV testing in TB patients after HIV testing guideline implementation in the UK	April 2008 - March 2009	Patients <18 years, private patients, on chemoprophylaxis, non TB mycobacteria were excluded.	TB patients in the database	HIV testing in the six months prior to and following TB notification	Men: 101 / 214 (47·2%)	NA	9/10
						Women: 76 / 193 (39·4%)		
Other publication types***								
**Reference (year)**	**Design and setting**	**Included study period**	**Population and exclusion criteria**	**IC definition**	**HIV tested definition**	**HIV test ratio (%)**	**Positivity ratio (%)**	
Aguayo (2010) [48]	Retrospective study on Extrapulmonary TB in Spain	NA	NA	Extrapulmonary tuberculosis	NA	11/20 (55·0%)	NA	
Hubbard (2020) [49]	Retrospective study on HBV and HCV prevalence in TB in a US hospital	September 2016 - May 2019	Adult cases of active or latent TB	positive QuantiFERON-TB Gold In-Tube test	Tested for HIV	375/453 (82·8%)	22/375 (5·9%)	
Patel (2019) [50]	Retrospective study on mortality risk factors and delays in TB mortality cases in New Mexico, US	2007 - 2017	NA	TB mortality cases	Offered HIV testing	48/83 (57·8%)	NA	
Perch (2013) [51]	Retrospective cohort on HIV testing in TB in Denmark	2009 (Total study included 2007–2009)	Notified TB of all ages cases were included	All cases of notified tuberculosis in database	NA	204/324 (63·0%)	8/204 (3·9%)	
Phillips (2010) [52]	Audit of IC-guided testing in UK hospital	October 2008 - November 2009	Patients of all ages with tuberculosis	Confirmed mycobacterium tuberculosis	HIV tested within the audit period	1/11 (9·1%)	NA	
Potter (2014) [53]	Audit on HBV, HCV and HIV infection among new TB cases in UK	2013	Patients of all ages with active TB	Active tuberculosis	HIV screened	447/472 (94·7%)	15/447 (3·4%)	
Qasim (2012) [54]	Audit on diagnosis and management of TB patients in the UK	January 2009 - December 2010	Patients with a positive Acid-Fast Bacillus test	A positive Acid-Fast Bacillus test	NA	21/21 (100%)	NA	
Reina (2015) [55]	Cross sectional study on unknown HIV status in TB patients in Portugal	2006 - 2012	TB cases reported	Registered tuberculosis cases	Known HIV status	6804/7683 (88·8%)	NA	
Ricci (2010) [56]	Audit on HIV testing and coinfection in TB patients in Italy	2004 - 2009	Patients with tuberculosis	Culture-confirmed cases of tuberculosis	Tested for HIV at any time	412/526 (78·3%)	67/412 (16·3%)	
Stolagiewicz (2015) [57]	Audit to quantify the local prevalence of HIV in patients with TB in the UK	2014	Patients diagnosed with or treated for TB	Diagnosed or treated for tuberculosis	Tested for HIV	114/114 (100%)	3/114 (2·6%)	
Thorburn (2012) [58]	Audit on HIV testing in TB patients in the UK	2010 (before implementation multidisciplinary TB meeting)	Confirmed TB cases in 2010	Confirmed TB cases	HIV tested in the year before or after TB diagnosis	2010: 141/234 (60·3%)	2010: 7/141 (5·0%)	
		2011 (after implementation multidisciplinary TB meeting)	Confirmed TB cases in 2011			2011: 81/105 (77·1%)	2011: 2/81 (2·5%)	
Vas (2012) [59]	Audit on HIV testing in TB patients in a UK hospital	2009	Patients attending the chest clinic with TB	NA	Patients offered and accepted an HIV test	9/34 (26·5%)	NA	
**Hepatitis B**								
**Full-text articles**								
**Reference (year)**	**Design and setting**	**Included study period**	**Population and exclusion criteria**	**IC definition**	**HIV tested definition**	**HIV test ratio (%)**	**Positivity ratio (%)**	Risk of bias score*
Augusti (2016) [19]	Cross-sectional cohort in primary care, Spain	January 2010 - August 2012	Patients aged 16–65 years were included; known HIV positive patients excluded	Using either their ICD-10 codes or a positive laboratory result	HIV test within 4 months of diagnosis date	Men: 1792/6034 (29·7%)	Men: 27/1792 (1·5%)	9/10
						Women: 1058/3712 (28·5%)	Women: 8/1058 (0·8%)	
Gupta (2011) [35]	First audit of IC-guided testing in UK general hospital	First audit: August 2008 - July 2009	Patients of all ages testing positive for HBV	Patients with a positive hepatitis B surface antigen test	HIV testing was double checked using the electronic pathology records system and a separate database of HIV testing	First audit: 6/27 (22·2%)	First audit: NA	7/10
	*Re*-audit of IC-guided testing in UK general hospital	*Re*-audit: August 2009 - June 2010				*Re*-audit: 10/44 (22·7%)	*Re*-audit: NA	
Hallager (2018) [31]	Retrospective cohort study on HIV coinfection among HBV and HCV patients in 18 hospitals in Denmark	January 2002 - July 2015	Patients registered in the Danish hepatitis database of 16 years or older	Positive HBV surface antigen	HIV antibody/antigen tests performed before or within 6 months of database enrolment	2287 / 3091 (74·0%)	89/2287 (3·9%)	9/10
Other publication types***								
**Reference (year)**	**Design and setting**	**Included study period**	**Population and exclusion criteria**	**IC definition**	**HIV tested definition**	**HIV test ratio (%)**	**Positivity ratio (%)**	
Deshpande (2015) [60]	Retrospective study on HIV testing in patients on Tenofovir monotherapy in Australia	January 2014 - June 2014	Patients with HBV on Tenofovir	Medical record or pathology confirmed chronic HBV infection	HIV test recorded before start of Tenofovir monotherapy	72/157 (45·9%)	NA	
Ireland (2018) [61]	Retrospective cross-sectional study on HIV testing in HBV patients in the UK	2010 - 2014	Patients of 15 years or over with HBV. Patients with known HIV and diagnosed with HBV at antenatal services were excluded	Hepatitis B virus (HBV) surface antigen positive	HIV tested on the same day or within 6 months following HBV diagnosis	7315/16,086 (45·5%)	NA	
Lander (2014) [62]	Audit on HIV testing in HBV and HCV patients in a hepatitis clinic in the UK	September 2012 - August 2013	HBV patients in the clinic	NA	Uptake of HIV testing in the clinic during the audit time period	205/362 (56·6%)	NA	
Lynn (2014) [63]	Audit on HIV testing in HBV patients in the Rochester Epidemiology Project (REP) in the US	1994 - 2010	HBV patients in the REP cohort	NA	All HIV screening tests and their results	273/607 (45·0%)	NA	
Pavlides (2011) [64]	Audit on HIV testing in HBV patients in the UK	October 2008 - September 2009	HBV patients	HBV surface antigen positive	Whether these patients had an HIV test	63/99 (63·6%)	6/63 (9·5%)	
Perera (2011) [65]	Audit On HIV testing in HBV patients in the UK	NA	HBV patients in a teaching hospital	NA	HIV test performed	53/88 (60·2%)	4/53 (7·5%)	
Phillips (2010) [52]	Audit on HIV testing in indicator conditions in the UK	October 2008 - November 2009	HBV patients at one hospital	Confirmed HBV infection	HIV tests taken within the same time period as inclusion	2/32 (6·3%)	NA	
Su (2015) [66]	Audit on HBV treatment and care at an Asian health center in the US	2012	New patients presenting with chronic hepatitis B	NA	Offered HIV screening	362/385 (94·0%)	NA	
Vas (2012) [59]	Audit on HIV testing in HBV patients in a hospital in the UK	2009	Patients attending the gastroenterology clinic with HBV	NA	Patients offered and accepted an HIV test	2/25 (8·0%)	NA	
**Hepatitis C**								
**Full-text articles**								
**Reference (year)**	**Design and setting**	**Included study period**	**Population and exclusion criteria**	**IC definition**	**HIV tested definition**	**HIV test ratio (%)**	**Positivity ratio (%)**	Risk of bias score*
Augusti (2016) [19]	Cross-sectional cohort in primary care, Spain	January 2010 - August 2012	Patients aged 16–65 years were included; known HIV positive patients excluded	Using either their ICD-10 codes or a positive laboratory result	HIV test within 4 months of diagnosis date	Men: 1995/6333 (31·5%)	Men: 67/1995 (1·1%)	9/10
						Women: 828/3493 (23·7%)	Women: 18/828 (2·2%)	
Bolther (2014) [30]	Cross-sectional cohort at a university hospital and outpatient clinics in Denmark	1996 - 2011	HCV patients of all ages; Patients no longer registered at the clinic were excluded	Chronic HCV patients with HCV RNA positive test outcome	HIV screening performance within 180 days of the HCV diagnosis	360/624 (57·7%)	NA	9/10
Gupta (2011) [35]	First audit of IC-guided testing in UK general hospital	First audit: August 2008 - July 2009	Patients of all ages testing positive for HCV	Patients with a positive hepatitis C antibody test	HIV testing was double checked using the electronic pathology records system and a separate database of HIV testing	First audit: 18/93 (19·4%)	First audit: NA	7/10
	*Re*-audit of IC-guided testing in UK general hospital	*Re*-audit: August 2009 - June 2010				*Re*-audit: 5/72 (6·9%)	*Re*-audit: NA	
Hallager (2018) [31]	Retrospective cohort study on HIV coinfection among HBV and HCV patients in 18 hospitals in Denmark	January 2002 - July 2015	Patients registered in the Danish hepatitis database of 16 years or older	HCV-RNA before or within 6 months after enrolment in the database	HIV antibody/antigen tests performed before or within 6 months of enrolment in the database	4400/5305 (82·9%)	281/4400 (6·4%)	9/10
King (2019) [67]	Intervention study among patients with an IC admitted to an acute General Medicine Unit in Australia	July 2017 - October 2017	Patients recently HIV tested, known HIV positive and with an alternative explanation for the IC were excluded	Hepatitis C antibody positive	Pathology lab data	5 / 11 (45·5%)	NA	4/10
Other publication types***								
**Reference (year)**	**Design and setting**	**Included study period**	**Population and exclusion criteria**	**IC definition**	**HIV tested definition**	**HIV test ratio (%)**	**Positivity ratio (%)**	
Cowan (2020) [68]	Retrospective review of testing and care of HCV mono- and HIV co-infected patients in a US emergency department	June 2018 - December 2019	Patients aged 18 years or older with active HCV infection, triaged to the ED and able to provide consent for testing	HCV viral load positive	Known HIV status	386/427 (90·4%)	56/386 (14·5%)	
Fleischer (2018) [69]	Retrospective cohort on HIV testing in HCV patients in a US hospital	July 2015 - March 2017	Patients with hepatitis C	HCV antibody positive	HIV antibody tested at any time	252/445 (56·6%)	6/252 (2·4%)	
Gilbert (2011) [70]	Audit to evaluate HIV testing in Canada	2007 - 2009	Patients diagnosed with HCV	NA	Tested for HIV within 3 months of diagnosis	8183/15,981 (51·2%)	NA	
Ireland (2018) [61]	Retrospective cross-sectional study on HIV testing in HCV cases in the UK	2010 - 2014	Patients of 15 years or over with HCV. Patients with known HIV were excluded	HCV antibody positive	HIV tested on the same day or within 6 months following HCV diagnosis	14,587/32,114 (45·4%)	NA	
Lander (2014) [62]	Audit on HIV testing in HBV and HCV patients in a hepatitis clinic in the UK	September 2012 - August 2013	HCV patients in the clinic	NA	Uptake of HIV testing in the clinic during the audit time period	40/72 (55·6%)	NA	
Lynn (2014) [63]	Audit on HIV testing in HCV patients in the Rochester Epidemiology Project in the US	1994 - 2010	HCV patients	NA	All HIV screening tests and their results	553/965 (57·3%)	NA	
Oraka (2016) [71]	Retrospective cohort on prevalence of HIV testing among adults with HCV in the US	1999 - 2014	Patients with HCV aged 20–59 years	HCV RNA positive test result	Ever tested for HIV	248/384 (64·6%)	NA	
Pavlides (2011) [64]	Audit on HIV testing in HCV patients in the UK	October 2008 - September 2009	HCV patients	Positive hepatitis C antibody or PCR	Whether these patients had an HIV test	51/102 (50·0%)	6/51 (11·8%)	
Perera (2011) [65]	Audit on HIV testing in HCV patients in the UK	NA	HCV patients in a teaching hospital	NA	HIV test performed	40/92 (43·5%)	3/40 (7·5%)	
Phillips (2010) [52]	Audit on HIV testing in indicator conditions in the UK	October 2008 - November 2009	HCV patients at one hospital	Confirmed HCV infection	HIV tests taken within the same time period as inclusion	25/88 (28·4%)	NA	
Sterling (2017) [72]	Retrospective cohort on HIV testing in HCV patients in the PROP UP cohort in the US	NA	HCV patients on DAA therapy enrolled in the PROP UP study, not known HIV positive	NA	HIV tested at some point in their history or prior to initiating DAA therapy	472/756 (62·4%)	NA	
Tunney (2018) [73]	Audit on management of HCV patients in the UK	March 2012 - March 2017	HCV patients (acute, chronic or past resolved) at the clinic, not known HIV positive	NA	HIV status	23/35 (65·7%)	NA	
Vas (2012) [59]	Audit on HIV testing in HCV patients in a hospital in the UK	2009	Patients attending the gastroenterology clinic with HCV	NA	Patients offered and accepted an HIV test	1/29 (3·4%)	NA	
**Hepatitis B or C**								
**Full-text articles**								
**Reference (year)**	**Design and setting**	**Included study period**	**Population and exclusion criteria**	**IC definition**	**HIV tested definition**	**HIV test ratio (%)**	**Positivity ratio (%)**	Risk of bias score*
Cayuelas Redondo (2019) [34]	Prospective interventional study on IC-guided HIV testing with an electronic prompt in primary healthcare in Spain	2013 (pre intervention)	Patients aged 18–65, with no known HIV infection, with acute or chronic hepatitis B or C	NA	HIV infection	Pre intervention: 2/26 (7·7%)	NA	8/10
		July 2014 - May 2015 (intervention)				During intervention: 5/17 (29·4%)		
		June 2015 - May 2016 (post intervention)				Post intervention: 1/21 (4·8%)		
Raben (2015) [9]	Retrospective cohort on HIV testing in ICs in Europe	May 2013	Patients in participating centres, >18 and <65 years of age, not known HIV positive, presenting within the previous year/last 100+ patients	Patients with hepatitis B or C	Participating centres reviewed retrospectively how many patients presenting with the IC were tested for HIV	2325/2681 (86·7%)	23/2325 (1·0%)	2/10
Other publication types***								
**Reference (year)**	**Design and setting**	**Included study period**	**Population and exclusion criteria**	**IC definition**	**HIV tested definition**	**HIV test ratio (%)**	**Positivity ratio (%)**	
Adlington (2014) [32]	An audit of HIV testing in acute medical patients with HIV clinical indicator conditions in the UK	January 2012	Patients with hepatitis B or C	Hepatitis B or C, registered as ICD-10 code	Whether HIV test had been performed during admission	January 2012: 10/39 (25·6%)	NA	
		January 2013				January 2013: 7/41 (17·1%)		
**Cervical carcinoma or cervical intraepithelial neoplasia grade 2+**								
**Full-text articles**								
**Reference (year)**	**Design and setting**	**Included study period**	**Population and exclusion criteria**	**IC definition**	**HIV tested definition**	**HIV test ratio (%)**	**Positivity ratio (%)**	Risk of bias score*
Alldredge (2020) [74]	Retrospective cohort study on HIV screening in women with newly diagnosed invasive cervical cancer in a large comprehensive US gynecologic oncology practice	January 2007 - December 2017	Women >18 years with invasive cervical cancer were included; cervical dysplasia, non-cervical or recurrent cancer and presenting at another specialty were excluded	International Classification of Diseases codes 180.9 and C53.9 for invasive cervical cancer	HIV-1/2 antibody or 4th generation p24 antigen test undertaken within 12 months before diagnosis, or within 30 days of the encounter.	38 / 492 (7·7%)	0/38 (0%)	9/10
Augusti (2016) [19]	Cross-sectional cohort in primary care, Spain	January 2010 - August 2012	Patients aged 16–65 years were included; known HIV positive patients excluded	Using either their ICD-10 codes or a positive laboratory result	HIV test within 4 months of diagnosis date	15/615 (2·4%)	0/15 (0%)	9/10
Gupta (2011) [35]	First audit of IC-guided testing in UK general hospital	First audit: August 2008 - July 2009	Patients of all age with cervical intraepithelial neoplasia	Patients of all ages with a positive pathology sample for CIN II or III	HIV testing was double checked using the electronic pathology records system and a separate database of HIV testing	First audit: 2/146 (1·4%)	NA	7/10
	*Re*-audit of IC-guided testing in UK general hospital	*Re*-audit: August 2009 - June 2010				*Re*-audit: 4/340 (1·2%)		
Hwang (2015) [75]	Retrospective cohort on HIV testing in patients with cancer at the initiation of therapy at a large US comprehensive cancer center	January 2004 - April 2011	Patients treated at a large comprehensive cancer center. Patients on oral chemotherapy and enrolled in clinical trials were excluded	Patients with cervical cancer who received systemic cancer therapy	HIV-1/2 antibody test and/or confirmatory Western blot testing after registration at the center.	23 / 245 (9·4%)	0/23 (0%)	10/10
McGee-Avila (2020) [76]	Retrospective study on patterns of HIV testing and determinants of non-receipt of HIV testing among women with cervical cancer in the New Jersey Medicaid program, US	January 2012 - December 2014	Patients with cervical cancer aged 21–64 years. Cases identified postmortem, non-New Jersey residence at diagnosis and with previous primary cancer or known HIV positive were excluded.	Primary, histologically confirmed invasive cervical cancer	Tested at any point during the study period	78/242 (32·2%)	NA	10/10
					Tested 6 months before diagnosis to 6 months after diagnosis of cervical cancer	33/242 (13·6%)		
Raben (2015) [9]	Retrospective cohort on HIV testing in ICs in Europe	May 2013	Patients in participating centres, >18 and <65 years of age, not known HIV positive, presenting within the previous year/last 100+ patients	Patients with cervical cancer	Participating centres reviewed retrospectively how many patients presenting with the IC were tested for HIV	444/583 (76·2%)	1/444 (0·2%)	2/10
Other publication types***								
**Reference (year)**	**Design and setting**	**Included study period**	**Population and exclusion criteria**	**IC definition**	**HIV tested definition**	**HIV test ratio (%)**	**Positivity ratio (%)**	
Butler (2014) [77]	Retrospective cohort study on HIV testing in patients with CIN 2+ in the UK	July 2012 - June 2013	Patients with CIN2+ at colposcopy, not known to be HIV positive	Cervical intraepithelial neoplasia grade 2 and above at colposcopy	The most recent HIV test at the service prior to their attendance for colposcopy (last 3 years)	34/94 (36·2%)	NA	
Lebari (2012) [78]	Retrospective review of HIV testing in patients with AIDS defining malignancies in the UK	March 2007 - July 2011	Patients referred or initially diagnosed with cervical cancer	NA	Tested for HIV	1/64 (1·6%)	NA	
Mosimann (2014) [79]	Retrospective cohort study on HIV testing rates among patients treated for AIDS defining cancers and HL in Switzerland	January 2002 - July 2012	Patients aged ≥ 18 years treated for invasive cervical cancer	Invasive cervical cancer	HIV tested within 90 days before and 90 days after the cancer diagnosis date	6/57 (10·5%)	0/6 (0%)	
**Malignant lymphoma**								
**Full-text articles**								
**Reference (year)**	**Design and setting**	**Included study period**	**Population and exclusion criteria**	**IC definition**	**HIV tested definition**	**HIV test ratio (%)**	**Positivity ratio (%)**	Risk of bias score*
Augusti (2016) [19]	Cross-sectional cohort in primary care, Spain	January 2010 - August 2012	Patients aged 16–65 years were included; known HIV positive patients excluded	Using either their ICD-10 codes or a positive laboratory result	HIV test within 4 months of diagnosis date	Patients with HL 0/86 (0%)	Patients with HL NA	9/10
						Men with NHL 6/250 (2·4%)	Men with NHL 1/6 (16·7%)	
						Women with NHL 6/214 (2·8%)	Women with NHL 0/6 (0%)	
Gupta (2011) [35]	First audit of IC-guided testing in UK general hospital	First audit: August 2008 - July 2009	Patients of all ages with lymphoma	Patients with a positive pathology sample for lymphoma	HIV testing was double checked using the electronic pathology records system and a separate database of HIV testing	First audit: 3/42 (7·1%)	NA	7/10
	*Re*-audit of IC-guided testing in UK general hospital	*Re*-audit: August 2009 - June 2010				*Re*-audit: 2/46 (4·3%)		
Hwang (2015) [75]	Retrospective cohort on HIV testing in patients with cancer at the initiation of therapy at a large US comprehensive cancer center	January 2004 - April 2011	Patients treated at a large comprehensive cancer center. Patients on oral chemotherapy and enrolled in clinical trials were excluded	Patients with NHL on systemic cancer therapy	HIV-1/2 antibody test and/or confirmatory Western blot testing after registration at the center.	NHL: 1439/1628 (88·4%)	NHL: 23/1439 (1·6%)	10/10
				Patients with HL on systemic cancer therapy		HL: 322/356 (90·4%)	HL: 2/322 (0·6%)	
Raben (2015) [9]	Retrospective cohort on HIV testing in ICs in Europe	May 2013	Patients in participating centres, >18 and <65 years of age, not known HIV positive, presenting within the previous year/last 100+ patients	Patients with NHL	Participating centres reviewed retrospectively how many patients presenting with the IC were tested for HIV	577/1274 (45·3%)	21/577 (3·6%)	2/10
Other publication types***								
**Reference (year)**	**Design and setting**	**Included study period**	**Population and exclusion criteria**	**IC definition**	**HIV tested definition**	**HIV test ratio (%)**	**Positivity ratio (%)**	
Bishin (2017) [80]	Longitudinal cohort study to assess treatment guidelines for diffuse large B-cell lymphoma in the US	2005 - 2016	All patients diagnosed and treated for diffuse large B-cell lymphoma	Diffuse large B-cell lymphoma	HIV serology testing	165/179 (92·2%)	NA	
Bowman (2010) [81]	Cohort study on HIV testing in lymphoma patients in the UK	6 month pilot period (date not reported)	All lymphoma patients seen in the 6 month pilot period at the study site	NA	NA	27/214 (12·6%)	0/27 (0%)	
Buxton (2011) [82]	Cross-sectional study to assess treatment in lymphoma patients in the UK	2009	All patients newly diagnosed with lymphoma	New lymphoma diagnosis	NA	91/281 (32·4%)	3/91 (3·3%)	
Datta (2015) [83]	Audit on treatment in Primary Central Nervous System lymphoma patients in the UK	2008 - 2013	All patients with Primary Central Nervous System lymphoma, excluding metastatic disease	Biopsy-proven Primary Central Nervous System lymphoma	HIV status	1/20 (5%)	NA	
Davies (2018) [84]	Audit on HIV testing in lymphoma patients in the UK	2016 - 2017	All patients newly diagnosed with lymphoma	New lymphoma diagnosis	Tested for HIV at first clinic/specialist review	101/135 (74·8%)	0/101 (0%)	
Lebari (2012) [78]	Retrospective review of HIV testing in patients with AIDS defining malignancies in the UK	March 2007 - July 2011	Patients referred or initially diagnosed with Non-Hodgkin's lymphoma	NA	Tested for HIV	34/158 (21·5%)	NA	
Mosimann (2014) [79]	Retrospective cohort study on HIV testing rates among patients treated for AIDS defining cancers and HL in Switzerland	January 2002 - July 2012	Patients aged ≥ 18 years treated for HL	Hodgkin's Lymphoma	HIV tested within 90 days before and 90 days after the cancer diagnosis date	HL: 79/133 (59·4%)	HL: 0/79 (0%)	
			Patients aged ≥ 18 years treated for NHL	non-Hodgkin lymphoma		NHL: 392/653 (60·0%)	NHL: 4/392 (1·0%)	

* Risk of bias was assessed for all included full-text references using an adapted version of the Joanna Briggs Institute checklist for prevalence studies, and scored out of 10. A risk of bias score of ≥7/10 was considered low risk by the researchers.

** If articles reported data on HIV test ratio and positivity ratio by subgroup (e.g. sex, before and after intervention), then the data of that article are provided by subgroup here.

*** Including abstracts, short communications, and correspondence.

CIN = cervical intraepithelial neoplasia; DAA = direct-acting antivirals; ED = emergency department; HBV = hepatitis B virus; HCV = hepatitis C virus; HL = Hodgkin's lymphoma; IC = indicator condition; ICD-10 = 10th revision of the International Classification of Diseases and Related Health Problems; NA = not reported/not applicable; NHL = Non-Hodgkin lymphoma; PCR = Polymerase chain reaction; REP = Rochester Epidemiology Project; RNA = ribonucleic acid; TB = tuberculosis.

### 3.2 Hepatitis B and C

Of three full-text references on HBV, one was performed in the PC setting, and the other two in hospitals. Median number of subjects was 3091 (IQR 71–9746). HIV test ratios were 23% and 74% in the hospital setting, and 29% in the PC setting. Median positivity was 2·6% (IQR 1·2%−3·9%). No studies were scored high risk of bias. Across the nine included other publications, median number of subjects was 157 (IQR 88–385) and median HIV test ratio was 46% (IQR 45%−60%).

Of five full-text references on HCV, one was performed in the PC setting, and the others in hospitals. Median number of subjects was 624 (IQR 165–5305). HIV test ratios ranged from 14% to 83% in the hospital setting, and was 29% in the PC setting. Median positivity was 4·7% (IQR 3·0%−6·4%). One study was scored high risk of bias. Across the 13 included other publications, median number of subjects was 384 (IQR 88–756) and median HIV test ratio 56% (IQR 45%−62%).

Two full-text references and one abstract did not distinguish between HBV and HCV. The full-text studies reported HIV test ratios of 13% and 87%, the abstract reported 21%, Table 2.

### 3.3 Cervical carcinoma or CIN2+

Of six full-text references on CC/CIN2+, one was performed in the PC setting, one in the context of a cancer surveillance program, and four in hospitals. Median number of subjects was 489 (IQR 245–583). The HIV test ratio was 2% in the PC setting. HIV test ratios ranged from 1% to 14% in four studies in the hospital/surveillance setting, and the fifth reported 76%, but risk of bias was deemed high. Of four studies reporting on positivity, three reported 0% and one 0·2% positivity. Three other publications were included, with a median number of 64 subjects (IQR 57–94) and median HIV test ratio of 11% (range 2%−36%), Table 2.

### 3.4 Malignant lymphoma

Of four full-text references on malignant lymphoma, one was performed in the PC setting, the others in hospitals. Median number of subjects was 869 (IQR 276–1629). HIV test ratios ranged from 6% to 89% in the hospital setting, and was 3% in the PC setting. Median positivity percentage was 3·6% (IQR 1·4%−8·3%). One study was high risk of bias. Across seven included other publications, median number of subjects was 179 (IQR 135–281) and median HIV test ratio was 32% (IQR 13%−75%), Table 2.

### 3.5 Pooled results

**Fig. 2:**
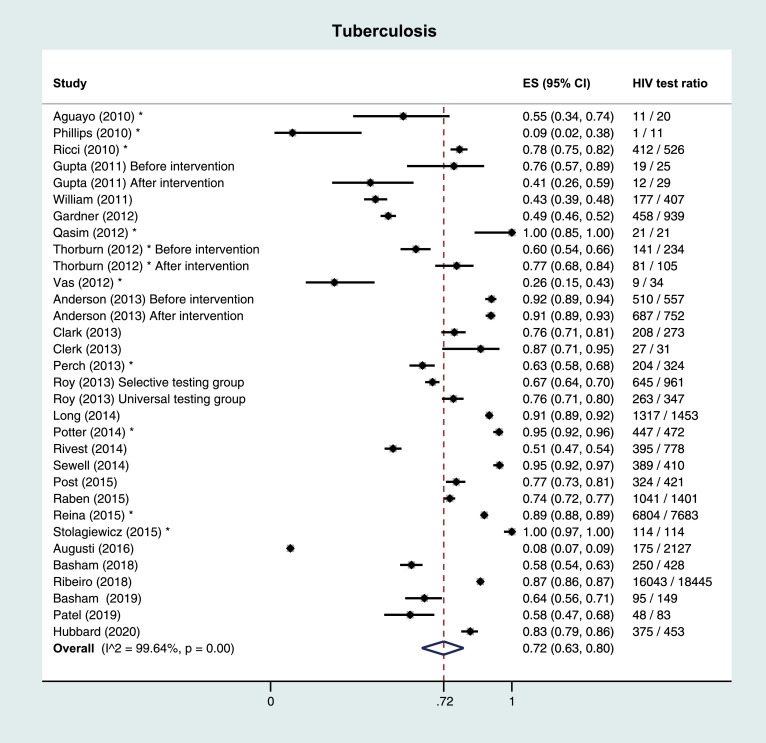

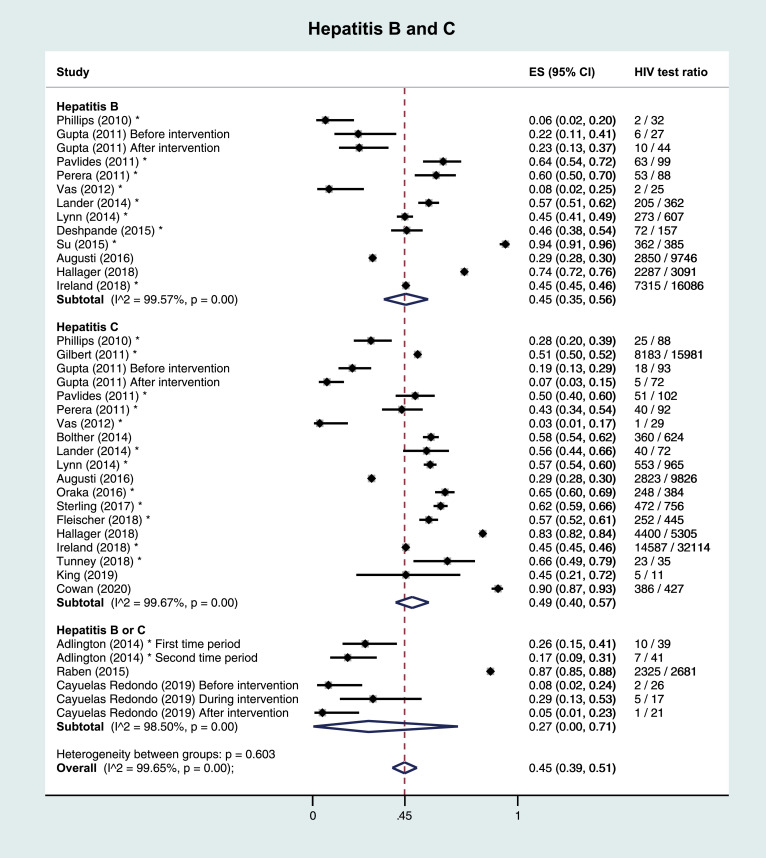

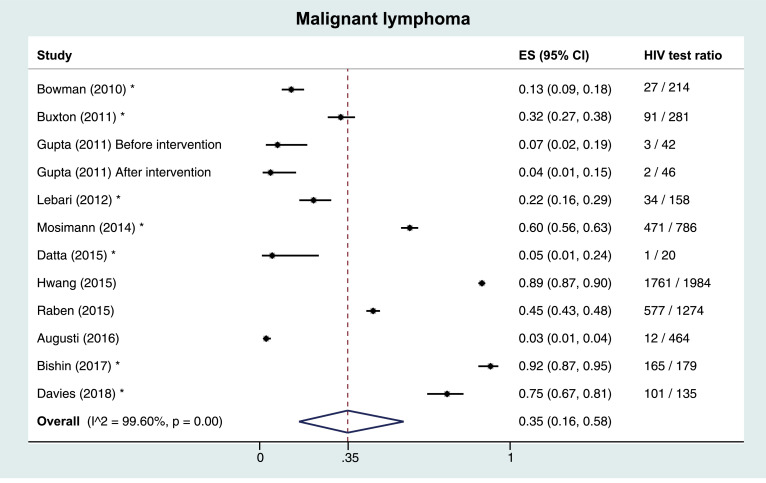

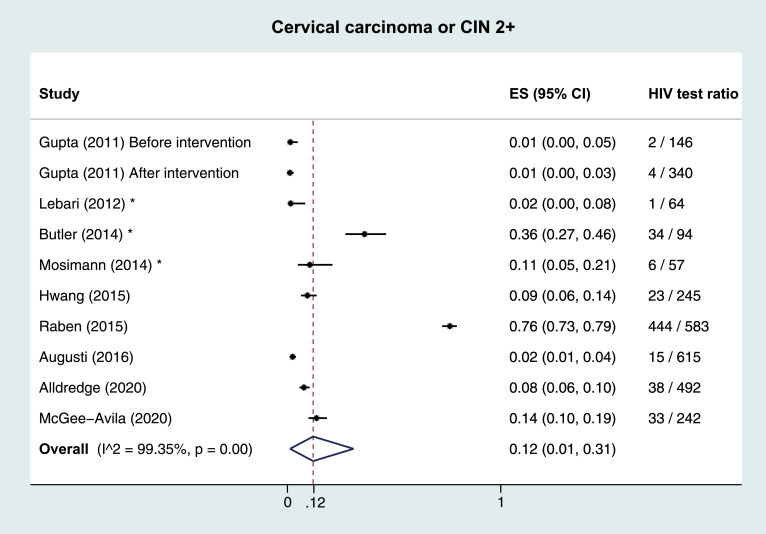
Pooled results and estimated proportion tested for HIV per indicator condition 2A: Tuberculosis 2B: Hepatitis B and C 2C: Malignant lymphoma 2D: Cervical carcinoma or cervical intraepithelial neoplasia grade 2+ * Other publication types than full-text articles (i.e. *abstracts, short communications, and correspondence). CIN: cervical intraepithelial neoplasia. ES: estimated proportion.*

Meta-analyses of HIV test ratios by IC, including all publication types, regardless of risk of bias were performed. Heterogeneity between studies within ICs was very large, with the I^2^ test for heterogeneity exceeding 99% in all analyses. The overall estimated proportion (ES) tested for HIV was 0·49 (95% CI 0·43–0·54). By IC, this proportion was highest in TB; ES 0·72 (0·63–0·80), followed by HCV (ES 0·49, 0·40–0·57), HBV (ES 0·45, 0·35–0·56), malignant lymphoma (ES 0·35, 0·16–0·58) and studies reporting hepatitis B or C (ES 0·27, 0·0–0·71). Lowest ES were observed in CC/CIN2+ (ES 0·12, 0·01–0·31), Fig. 2. A sensitivity analysis including only low risk of bias full-text publications showed lower proportions, with an overall ES of 0·40 (0·29–0·52), and 0·68 (0·51–0·83), 0·38 (0·10–0·71), 0·37 (0·10–0·69), 0·21 (0·00–0·87), 0·12 (0·02–0·27), and 0·05 (0·02–0·09) for TB, HCV, HBV, malignant lymphoma, HBV/HCV, and CC/CIN2+, respectively. Five studies reported HIV test ratios stratified by sex; four on TB, and one on TB, HBV, HCV, and malignant lymphoma. When pooling studies among TB patients by sex, overall ES were similar in women (ES 0·49, 0·13–0·85) and men (ES 0·53, 0·15–0·89).

Meta-analyses of HIV positivity by IC, including all publication types, regardless of risk of bias were performed. Heterogeneity between studies within ICs was large for most ICs (e.g. I^2^ test for heterogeneity 96% for HBV/HCV), but low for CC/CIN2+ (I^2^=0%). The overall estimated positivity ranged between 0% (CC/CIN2+) and 5% (TB) (**supplementary appendix 6**).

Meta-regression analyses showed no significant association between date of data collection and overall HIV test ratio (β=1·05%, 95%CI=−0·96%–3·06%, *p* = 0·30). When stratified by IC, a significant association was observed in studies on CC/CIN2+ only (β=6·14%, 95%CI=0·75%–11·53%, *p* = 0·03). However, this association was largely influenced by the most recent study, that reported the highest test ratio, but was also deemed high risk of bias. [9] In a sensitivity analysis excluding high risk of bias studies, this association was lost (β=0·47%, 95%CI=−2·86%–3·79%, *p* = 0·72).

## 4 Discussion

This systematic review provides an overview of the adoption of IC-guided testing in seven selected ICs in Western countries. Results show a large variation in HIV test ratios per IC, but overall HIV test ratios are low. The highest test ratios were observed in TB patients, followed by patients with HCV, HBV, and malignant lymphoma, respectively. Lowest test ratios were observed in patients with CC/CIN2+. No data on the extent of IC-guided testing in patients with VC/VIN2+ and PN was found.

Large differences in HIV test ratios between studies concerning the same IC were observed. Some outliers were studies with a high risk of bias, but among studies with low risk of bias, considerable variation was still observed. An explanation may be the difference in design of studies and how being tested for HIV was defined: some studies assessed evidence of any HIV testing, while others had a set timeframe around IC diagnosis to assess IC-guided testing. Another explanation could be the difference in setting between studies. For example, in malignant lymphoma, the lowest test ratio was observed in a study performed in the PC setting, while the highest ratio was observed in a study performed in a comprehensive cancer center. Variation was also observed within countries. Among TB patients in Canada, an audit performed in the province of Manitoba showed much lower HIV test ratios than one in Alberta (59% versus 91%, respectively [28,29]). This discrepancy is probably due to the ‘opt out’ HIV testing procedure for TB patients in Alberta, which was not used in Manitoba, suggesting its effectivity to optimize HIV testing. Two studies performed among HCV patients in Denmark also showed very different results, with an HIV test ratio of 58% in one university hospital, [30] compared to 83% in 18 Danish hospitals. [31] This discrepancy might be due to an increase in HIV test ratio over time, attributed to national efforts to increase HIV testing in risk groups, as the latter study concerned a later period (2002–2015) than the former (1996–2011). However, we found no association between data collection period and HIV test ratio in meta-regression analyses, suggesting that adherence to this testing strategy has not improved over time, and underlining the urgency of implementing strategies to improve IC-guided testing for HIV.

When comparing HIV test ratio before- and after interventions to increase HIV testing, some studies reported an improvement, [32,33] while others did not. [34,35] One UK study showed that HIV test ratios among patients with TB, HCV, cervical carcinoma and malignant lymphoma were lower in 2009–2010 than in 2008–2009 despite educational and promotional efforts by the researchers. [35] Studies showed that a universal HIV testing policy among TB patients yielded higher HIV test ratios than a selective testing policy based on risk-assessment, [36] a result in line with the high success rate of the ‘opt out’ testing procedure for TB patients in Alberta. [29]

HIV positivity was highest among TB patients, followed by HBV, HCV, and malignant lymphoma, respectively, but again large variation was observed. Among CC/CIN2+ patients, one study reported a positivity of 0·2%, [9] while positivity was 0% in the others. However, in view of the small number of studies and the low test ratios, this should not be interpreted as HIV screening not being cost-effective among CC/CIN2+ patients [4,5.]

This review confirms previous reporting on missed opportunities for earlier diagnosis through IC-guided testing. A barrier to optimal IC-guided testing might be the large number of ICs, the large variety in types of conditions and the many medical specialties involved. This variety requires tailored strategies to assure routine IC-guided testing is implemented across ICs. Moreover, an evaluation of IC-guided HIV testing recommendations in specialty guidelines in the UK and Europe revealed that the majority of IC guidelines do not recommend HIV testing, and physicians are not always aware of current HIV testing recommendations. [18,37] This is supported by the observation that the highest HIV test ratio were found in TB, HBV and HCV; HIV testing is recommended most prominently in the specialty guidelines for these conditions, and as pulmonologists and gastroenterologists commonly collaborate with infectious disease specialists, they may be more likely to focus on possible underlying HIV. Adoption of HIV testing in specialty guidelines and creating awareness of this strategy among involved specialties is an important first step to optimize testing. [38] As educational interventions to optimize testing showed varying results, additionally implementing previously proven successful strategies, such as opt-out testing or universal testing without detailed pre-test discussion, as described in the studies mentioned earlier, [29,36] is likely more effective than only educating involved medical professionals on IC-guided testing. In addition, sustained effect must be aimed for when designing interventions. For example, a digital case note prompt suggesting HIV testing when the patient has an IC diagnosis lead to a significant increase in HIV test ratios during the intervention period in two studies, but the effect was lost when the prompts were deactivated. [24,34] Thus, continuous implementation of a combination of the aforementioned strategies would likely be most effective.

A major strength of this review is the variety of settings and countries included. Second, by including not only published full-text articles, but also other publication types, we gained a more comprehensive picture of actual IC-guided HIV testing practices.

Although the retrospective design of included studies posed a potential risk of bias, most full-text studies were assessed as low risk of bias. We further addressed this possible limitation in a sensitivity analysis including only full-text articles with low risk of bias and found lower estimated proportions, suggesting that the IC-guided HIV test ratio outside study settings might be even lower. Very large heterogeneity was observed in the meta-analyses by IC, probably reflecting true heterogeneity across settings and Western countries. Thus, exact inferences on HIV test ratios by ICs could not be made, but conclusions can be drawn from the heterogeneity itself; testing practices are both inconsistently reported and inconsistently adopted. These findings should be considered when evaluating efforts to improve HIV testing strategies. Finally, a selection of only seven ICs was included in this review. Although not all ICs were included, it is unlikely that the HIV test ratios in other ICs will be much higher, as well-established and guideline-supported ICs such as TB and HCV were included in this study, and it is evident that even in those improvement is still needed.

This systematic review shows that a decade after its introduction, IC-guided testing for HIV is still insufficiently implemented in Western countries. Lessons on effective strategies from ICs with the highest test ratios, such as universal testing strategies, should be used to design effective implementation strategies for optimal IC-guided testing, to reduce underdiagnosis and late presentation of HIV.

## Funding

The H-TEAM initiative, of which this review is a project, is being supported by Aidsfonds (Grant No. 2013169), Stichting Amsterdam Dinner Foundation, Bristol-Myers Squibb International Corp. (study number: AI424–541), Gilead Sciences Europe Ltd (Grant No. PA-HIV-PREP-16-0024), Gilead Sciences (protocol numbers: CO-NL-276-4222, CO-US-276-1712), Janssen Pharmaceutica (reference number: PHNL/JAN/0714/0005b/1912fde), M.A.C AIDS Fund, ViiV Healthcare (PO numbers: 3000268822, 3000747780) and ZonMw (Grant No. 522002003). This review is further funded by Aidsfonds (Grant No. P- 42702). The funders of the study had no role in the study design or execution.

## Data sharing statement

The datasets generated and/or analysed during the current study are available from the corresponding author upon reasonable request.

## Contributors statement

Geerlings acquired financial support for the project leading to this publication. Geerlings, Schim van der Loeff, van Bergen and Bogers were involved in the conceptualisation and design of methodology of the study. Bogers performed the literature search. Bogers and Hulstein performed data curation including screening of search results, data extraction, and performing quality assessments. Bogers analysed the data and designed the figures. Schim van der Loeff performed quality controls and validation on all data analyses. De Bree and Reiss provided commentary and revisions on the original draft. All authors were involved in the interpretation of the data and the preparation of the final manuscript.

## Declaration of Competing Interest

Dr. Bogers reports grants from Aidsfonds, grants from H-TEAM, during the conduct of the study; and this work is partially funded by H-TEAM, a consortium of all actors involved in hiv-care and prevention in Amsterdam, with the ultimate goal to pursue the end of new hiv-infections in Amsterdam. The H- team is sponsored by a mix of organisations, including the Municipality, AIDS-funds, and several farmaceutical companies (www.hteam.nl). There are no personal fees or payment involved. H-TEAM is not involved in the design or conduct of this work. S.H.H. Hulstein has nothing to disclose. Dr. Schim van der Loeff reports other from Merck, non-financial support from Stichting Pathologie Onderzoek en Ontwikkeling (SPOO), outside the submitted work. Dr. de Bree reports grants from AIDS Fonds, grants from Stichting AmsterdamDiner Foundation, grants from Gilead Sciences, grants from Janssen Pharmaceutica, grants from ViiV Healthcare, grants from ZonMW, grants from M.A.C AIDS Fund, during the conduct of the study; grants and other from Gilead Sciences, outside the submitted work. Dr. Reiss reports grants from AIDS Fonds, grants from Stichting AmsterdamDiner Foundation, grants from Gilead Sciences, grants from Janssen Pharmaceutica, grants from ViiV Healthcare, grants from ZonMW, grants from M.A.C AIDS Fund, during the conduct of the study; grants and other from Gilead Sciences, grants and other from ViiV Healthcare, grants and other from Merck, outside the submitted work. Dr. van Bergen reports grants from RIVM: national institute for public health and the environment, during the conduct of the study; and reports being a member of the board of the H-TEAM, a consortium of all actors involved in hiv-care and prevention in Amsterdam, with the ultimate goal to pursue the end of new hiv-infections in Amsterdam. The H- team is sponsored by a mix of organisations, including the Municipality, AIDS-funds, and several farmaceutical companies (www.hteam.nl). There are no personal fees or payment involved. Dr. Geerlings reports grants from Aidsfonds, during the conduct of the study.

## Appendix Supplementary materials

Image, application 1
