# Supplementary material for: Current evidence on the adoption of indicator condition guided testing for HIV in western countries: A systematic review and meta-analysis
Source: eClinicalMedicine. 2021 May 8;35:100877. doi: 10.1016/j.eclinm.2021.100877 (PMC8129933; doi:10.1016/j.eclinm.2021.100877)
Supplement: Supplementary file 1 [file mmc1.docx]

**Online supplementary materials to:**

**Current evidence on the adoption of indicator condition guided testing for HIV in Western Countries: A systematic review and meta-analysis**

Appendix 1: PROSPERO study protocol

Appendix 2: PRISMA checklist for reporting Systematic Reviews and Meta-analyses

Appendix 3: Full search strategy

Appendix 4: Data extraction sheet

Appendix 5: Joanna Briggs Institute risk of bias checklist

Appendix 6: Figure: Meta-analyses of HIV positivity by indicator condition

Appendix 7: H-TEAM consortium members

## Appendix 1: PROSPERO study protocol

The protocol for this review was published at PROSPERO, registration number CRD42020160243:


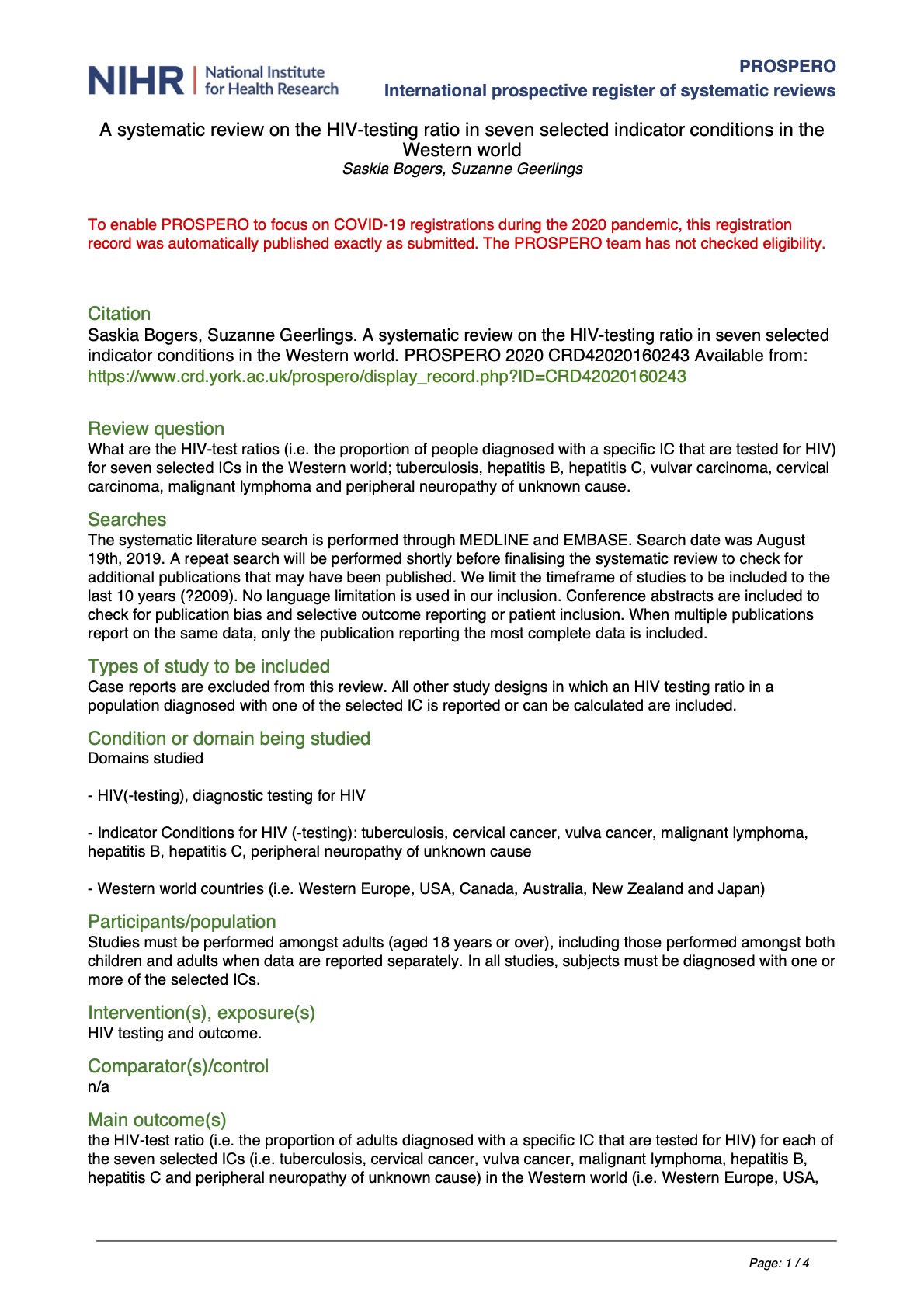


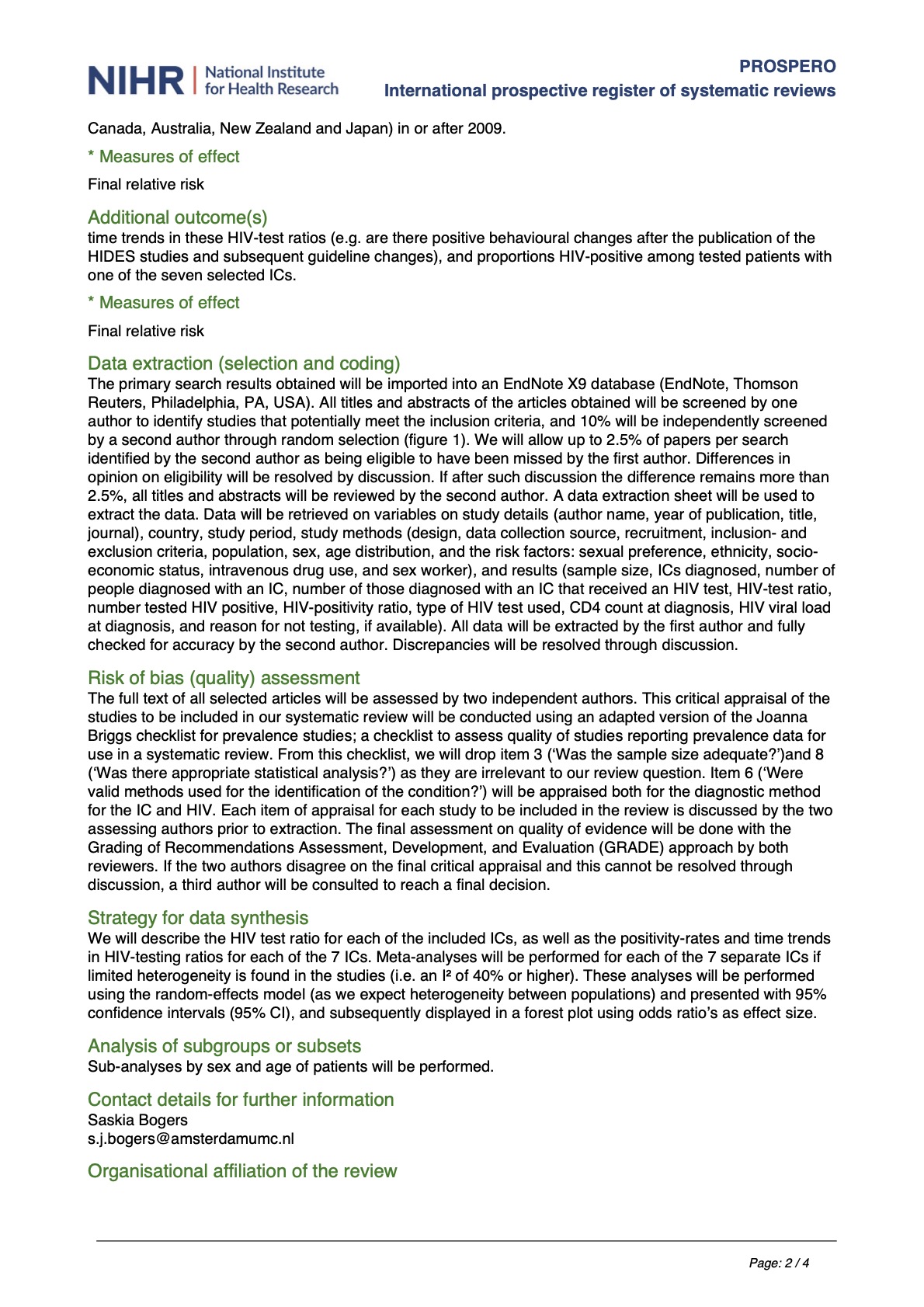

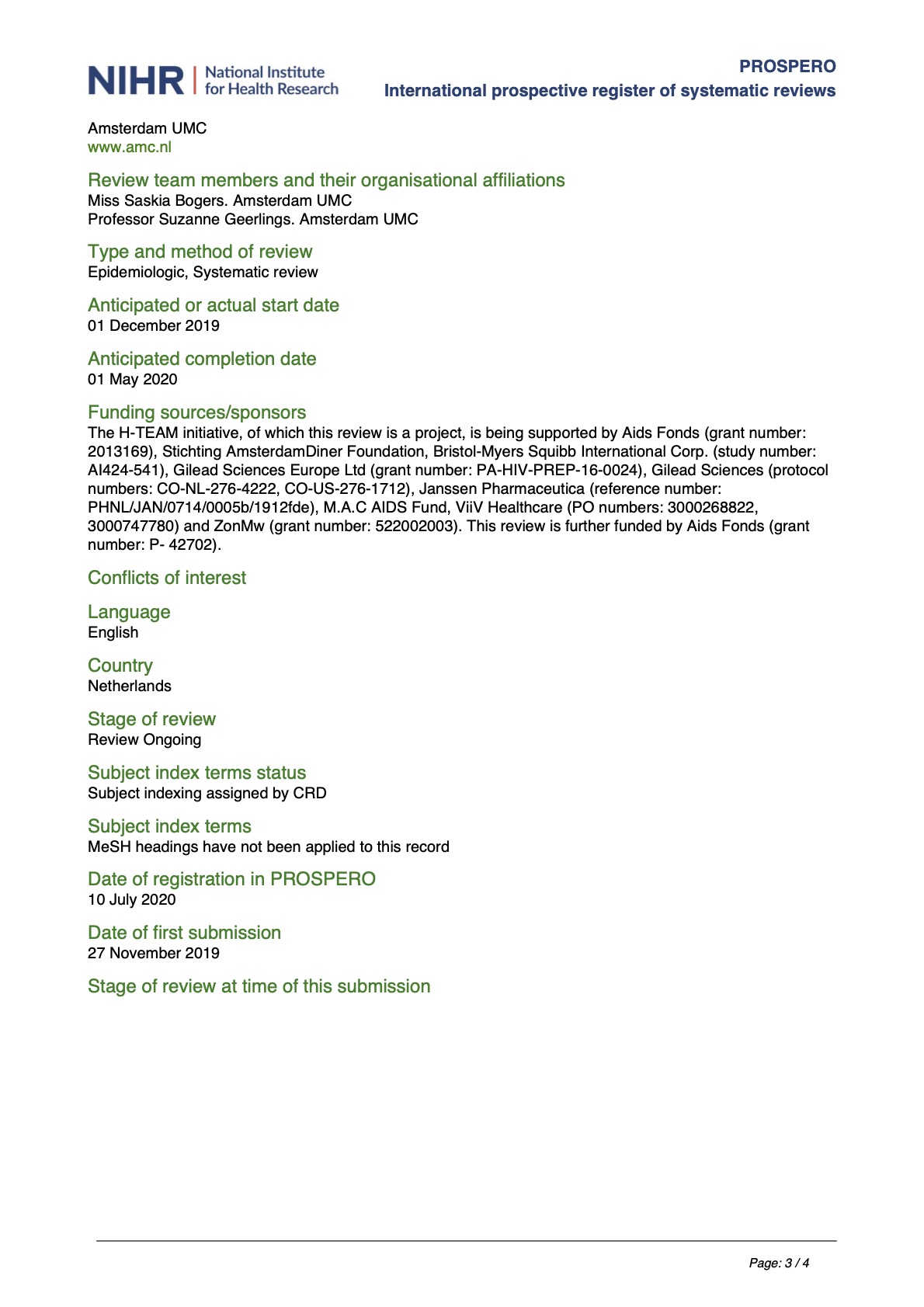

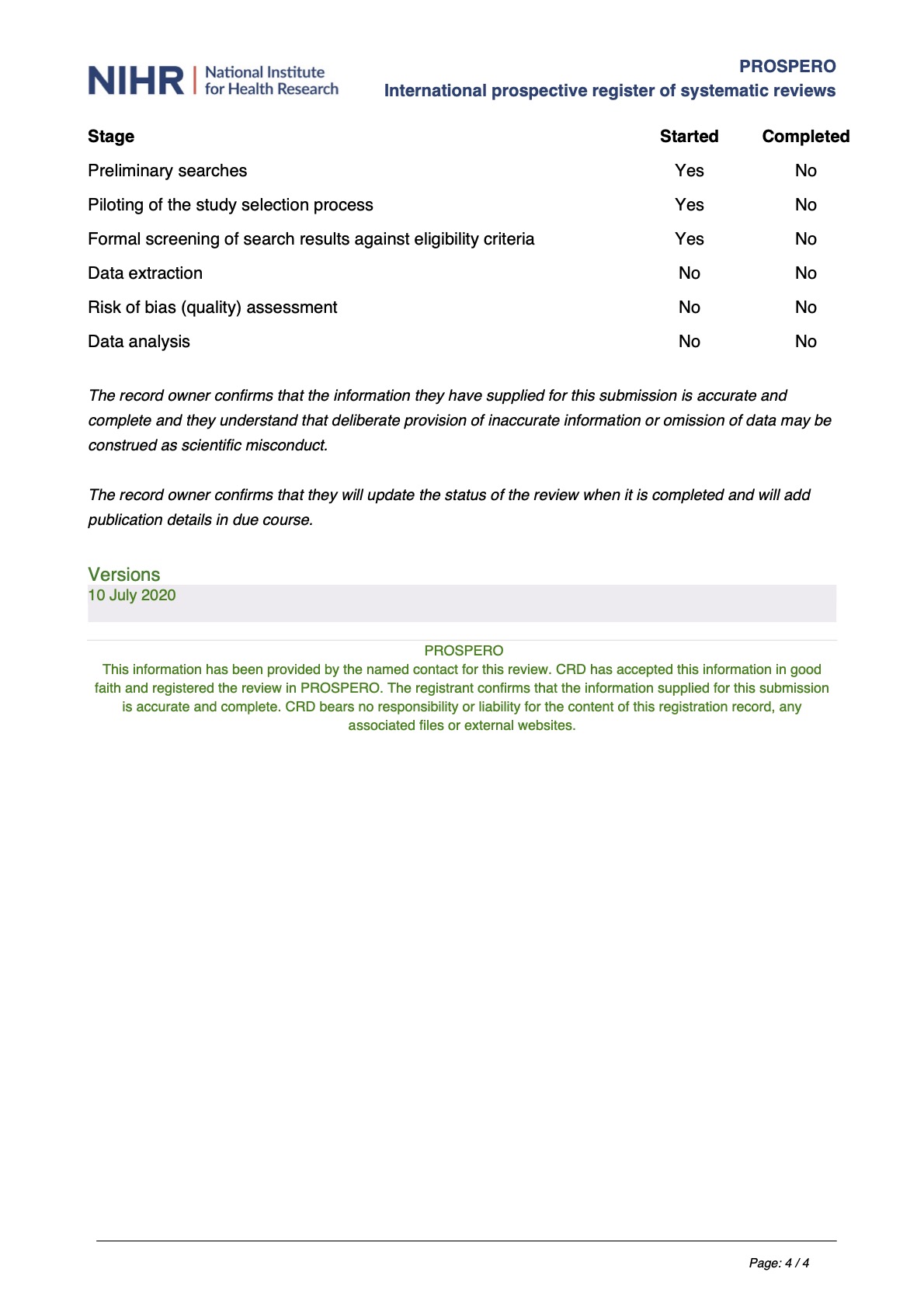


## Appendix 2: PRISMA checklist for reporting Systematic Reviews and Meta-analyses

| **Section/topic** | **#** | **Checklist item** | **Reported on page #** |
| --- | --- | --- | --- |
| **TITLE** | | |  |
| Title | 1 | Identify the report as a systematic review, meta-analysis, or both. | 1 |
| **ABSTRACT** | | |  |
| Structured summary | 2 | Provide a structured summary including, as applicable: background; objectives; data sources; study eligibility criteria, participants, and interventions; study appraisal and synthesis methods; results; limitations; conclusions and implications of key findings; systematic review registration number. | 2 |
| **INTRODUCTION** | | |  |
| Rationale | 3 | Describe the rationale for the review in the context of what is already known. | 3 |
| Objectives | 4 | Provide an explicit statement of questions being addressed with reference to participants, interventions, comparisons, outcomes, and study design (PICOS). | 3 |
| **METHODS** | | |  |
| Protocol and registration | 5 | Indicate if a review protocol exists, if and where it can be accessed (e.g., Web address), and, if available, provide registration information including registration number. | 4 |
| Eligibility criteria | 6 | Specify study characteristics (e.g., PICOS, length of follow-up) and report characteristics (e.g., years considered, language, publication status) used as criteria for eligibility, giving rationale. | 4 |
| Information sources | 7 | Describe all information sources (e.g., databases with dates of coverage, contact with study authors to identify additional studies) in the search and date last searched. | 4 |
| Search | 8 | Present full electronic search strategy for at least one database, including any limits used, such that it could be repeated. | 4, appendix page 4 |
| Study selection | 9 | State the process for selecting studies (i.e., screening, eligibility, included in systematic review, and, if applicable, included in the meta-analysis). | 5 |
| Data collection process | 10 | Describe method of data extraction from reports (e.g., piloted forms, independently, in duplicate) and any processes for obtaining and confirming data from investigators. | 5 |
| Data items | 11 | List and define all variables for which data were sought (e.g., PICOS, funding sources) and any assumptions and simplifications made. | 5, appendix page 5 |
| Risk of bias in individual studies | 12 | Describe methods used for assessing risk of bias of individual studies (including specification of whether this was done at the study or outcome level), and how this information is to be used in any data synthesis. | 5, 6, appendix page 6 |
| Summary measures | 13 | State the principal summary measures (e.g., risk ratio, difference in means). | 5-6 |
| Synthesis of results | 14 | Describe the methods of handling data and combining results of studies, if done, including measures of consistency (e.g., I^2^) for each meta-analysis. | 5-6 |
| Risk of bias across studies | 15 | Specify any assessment of risk of bias that may affect the cumulative evidence (e.g., publication bias, selective reporting within studies). | 5 |
| Additional analyses | 16 | Describe methods of additional analyses (e.g., sensitivity or subgroup analyses, meta-regression), if done, indicating which were pre-specified. | 6 |

Page 1 of 2

| **Section/topic** | **#** | **Checklist item** | **Reported on page #** |
| --- | --- | --- | --- |
| **RESULTS** | | |  |
| Study selection | 17 | Give numbers of studies screened, assessed for eligibility, and included in the review, with reasons for exclusions at each stage, ideally with a flow diagram. | 7, Figure 1, Table 1 |
| Study characteristics | 18 | For each study, present characteristics for which data were extracted (e.g., study size, PICOS, follow-up period) and provide the citations. | 7-8, Table 2 |
| Risk of bias within studies | 19 | Present data on risk of bias of each study and, if available, any outcome level assessment (see item 12). | 7-8, Table 2 |
| Results of individual studies | 20 | For all outcomes considered (benefits or harms), present, for each study: (a) simple summary data for each intervention group (b) effect estimates and confidence intervals, ideally with a forest plot. | 7-9, Table 2, Figure 2 |
| Synthesis of results | 21 | Present results of each meta-analysis done, including confidence intervals and measures of consistency. | 8-9 |
| Risk of bias across studies | 22 | Present results of any assessment of risk of bias across studies (see Item 15). | n/a  (see pg. 5) |
| Additional analysis | 23 | Give results of additional analyses, if done (e.g., sensitivity or subgroup analyses, meta-regression [see Item 16]). | 8-9 |
| **DISCUSSION** | | |  |
| Summary of evidence | 24 | Summarize the main findings including the strength of evidence for each main outcome; consider their relevance to key groups (e.g., healthcare providers, users, and policy makers). | 10 |
| Limitations | 25 | Discuss limitations at study and outcome level (e.g., risk of bias), and at review-level (e.g., incomplete retrieval of identified research, reporting bias). | 11-12 |
| Conclusions | 26 | Provide a general interpretation of the results in the context of other evidence, and implications for future research. | 10-12 |
| **FUNDING** | | |  |
| Funding | 27 | Describe sources of funding for the systematic review and other support (e.g., supply of data); role of funders for the systematic review. | 36 |

From: Moher D, Liberati A, Tetzlaff J, Altman DG, The PRISMA Group (2009). Preferred Reporting Items for Systematic Reviews and Meta-Analyses: The PRISMA Statement. *PLoS Med* **6(7)**: e1000097.

For more information, visit: www.prisma-statement.org

Page 2 of 2

## Appendix 3: Full search strategy

| **Ovid MEDLINE(R) ALL <1946 to November 20, 2020>** | | |
| --- | --- | --- |
| **#** | **Searches** | **Results** |
| 1 | exp Tuberculosis/ | 189990 |
| 2 | (tubercul* or tb or tbc).ti,ab,kf. | 252288 |
| 3 | 1 or 2 | 277322 |
| 4 | Uterine Cervical Neoplasms/ | 72306 |
| 5 | (cervi* adj5 (cancer* or neoplas* or carcinom* or malignan* or tumor* or tumour*)).ti,ab,kf. | 85841 |
| 6 | 4 or 5 | 104132 |
| 7 | exp Vulvar Neoplasms/ | 8033 |
| 8 | ((vulva* or clitoris or clitoral) adj3 (cancer* or neoplas* or carcinom* or malignan* or tumor* or tumour*)).ti,ab,kf. | 5955 |
| 9 | 7 or 8 | 9525 |
| 10 | exp Lymphoma/ | 167356 |
| 11 | (lymphoma* or hematologic-neoplasms* or hodgkin* or non-hodgkin* or nonhodgkin* or immuno?ytom* or (hair* adj cell* adj leu*) or burkit* or sezary* or (mycos* adj fungo*) or (h?emato* adj (malign* or neoplas*))).ti,ab,kf. | 219363 |
| 12 | 10 or 11 | 259698 |
| 13 | hepatitis b/ or hepatitis c/ | 73777 |
| 14 | (hepatitis or ((HBV or HCV) adj infection)).ti,ab,kf. | 214749 |
| 15 | 13 or 14 | 224454 |
| 16 | exp Peripheral Nervous System Diseases/ | 142614 |
| 17 | (neuropathy or neuropathies or polyneuropathies or polyneuropathy).ti,ab,kf. | 81375 |
| 18 | 16 or 17 | 187244 |
| 19 | (indicator adj (diseas* or condition*)).ti,ab. | 228 |
| 20 | 3 or 6 or 9 or 12 or 15 or 18 or 19 | 1043189 |
| 21 | (HIV adj2 (testing or screening)).ti,ab,kf. | 15936 |
| 22 | 20 and 21 | 1946 |
| 23 | case reports.pt. | 2038816 |
| 24 | 22 not 23 | 1886 |

| **Embase Classic + Embase <1947 to November 20, 2020>** | | |
| --- | --- | --- |
| **#** | **Searches** | **Results** |
| 1 | exp tuberculosis control/ or exp tuberculosis/ | 258284 |
| 2 | (tubercul* or tb or tbc).ti,ab,kw. | 311283 |
| 3 | 1 or 2 | 362256 |
| 4 | exp uterine cervix cancer/ | 98698 |
| 5 | (cervi* adj5 (cancer* or neoplas* or carcinom* or malignan* or tumor* or tumour*)).ti,ab,kw. | 119298 |
| 6 | 4 or 5 | 144077 |
| 7 | exp vulva cancer/ | 7040 |
| 8 | ((vulva* or clitoris or clitoral) adj3 (cancer* or neoplas* or carcinom* or malignan* or tumor* or tumour*)).ti,ab,kw. | 8698 |
| 9 | 7 or 8 | 11137 |
| 10 | exp lymphoma/ | 311845 |
| 11 | (LYMPHOMA* or HEMATOLOGIC-NEOPLASMS* or HODGKIN* or NON-HODGKIN* or NONHODGKIN* or IMMUNO?YTOM* or (HAIR* adj CELL* adj Leu*) or BURKIT* or SEZARY* or (MYCOS* adj FUNGO*) or (H?EMATO* adj (MALIGN* or neoplas*))).ti,ab,kw. | 318893 |
| 12 | 10 or 11 | 404124 |
| 13 | exp Hepatitis A virus/ or exp hepatitis A/ or exp hepatitis B/ or exp Hepatitis B virus/ | 144399 |
| 14 | (hepatitis or ((HBV or HCV) adj infection)).ti,ab,kw. | 310961 |
| 15 | 13 or 14 | 338643 |
| 16 | exp neuropathy/ | 554778 |
| 17 | (neuropathy or neuropathies or polyneuropathies or polyneuropathy).ti,ab,kw. | 125382 |
| 18 | 16 or 17 | 575735 |
| 19 | (indicator adj (diseas* or condition*)).ti,ab. | 370 |
| 20 | 3 or 6 or 9 or 12 or 15 or 18 or 19 | 1778570 |
| 21 | (HIV adj2 (testing or screening)).ti,ab,kw. | 20618 |
| 22 | 20 and 21 | 3195 |
| 23 | case report/ | 2491837 |
| 24 | 22 not 23 | 3001 |
| 25 | limit 24 to (conference abstracts or embase) | 2621 |
| 26 | limit 24 to embase | 1619 |
| 27 | limit 24 to conference abstracts | 1002 |

## Appendix 4: Items included in the data extraction sheet

Data extraction was performed using a standard data extraction form created in Microsoft Excel.

The items in the data extraction were:

| **Item** | **Further instruction** |
| --- | --- |
| First author name | Name of first author |
| Study # | Record number from title/abstract screening results |
| Indicator condition | Which IC is reported on. If multiple ICs are reported on, extract each IC in a separate entry |
| Lymphoma type | For lymphoma: Specify which type. Use the exact specification that the article used |
| Article or abstract | Is the reported data from an article or abstract |
| Year of publication | Publication year |
| Title | Title of the publication |
| Journal | Journal or conference publised/presented |
| Country/Countries | Country/countries in which the research took place |
| Study period | Period which was studied |
| Aim of study | Main research question |
| Recruitment site | Site of recruitment of patients/ data collection |
| Study design | Design of study |
| IC definition | How was the IC diagnosis defined |
| HIV tested definition | How was 'being tested for HIV' defined |
| Data collection source | Where was the data collected from (type of data source, e.g. registry, health records) |
| Inclusion criteria | What were the inclusion criteria |
| Exclusion criteria | What were the exclusion criteria |
| Population | Which population was studied (for the data collected in this row of the form, e.g. only the women in one row, and only the men in the next for the same study) |
| Age (describe what is reported, mean, median, ranges, categories) | Descriptive reporting of age distribution (as described in paper) |
| # with IC | Number of subjects with the IC, population in this row |
| # with HIV test | Number of subjects that were tested for HIV |
| HIV test ratio (calculation) | Calculation column for HIV test ratio |
| Reported HIV test ratio | HIV test ratio as reported in the record |
| # positive HIV tests | Number of population that were tested that were positive |
| Calculated HIV positivity ratio | Calculation column for HIV positivity ratio |
| Reported HIV positivity ratio | HIV positivity ratio as reported in article |
| Type of HIV test used | Type of HIV test used in the study |
| CD4 count at diagnosis | With unit that was used in the article |
| Viral load at diagnosis | With unit that was used in the article |
| Reasons for not testing | If reported |
| Author's comments | Additional comments by authors of record |
| Reviewer comments | Additional comments of screener |

IC: Indicator condition.

## Appendix 5: Joanna Briggs Institute risk of bias checklist^1^

For assessment of risk of bias of individual included full-text studies, an adapted version of the Joanna Briggs Institute critical appraisal checklist for studies reporting prevalence data was used;

The original checklist contains the following items:

1. Was the sample representative of the target population?
2. Were study participants recruited in an appropriate way?
3. Was the sample size adequate?
4. Were the study subjects and setting described in detail?
5. Is the data analysis conducted with sufficient coverage of the identified sample?
6. Were objective, standard criteria used for measurement of the condition?
7. Was the condition measured reliably?
8. Was there appropriate statistical analysis?
9. Are all important confounding factors/ subgroups/differences identified and accounted for?
10. Were subpopulations identified using objective criteria?

For our risk of bias assessment, we dropped item 8 of the checklist, as it was not deemed relevant for our study question. We assessed item 6 for both the indicator condition that was studied, as well as how ‘tested for HIV’ was assessed. Thus, the total number of items was 10.

Scoring of all full-text articles was done by two authors (SJB and SHH) independently and any discrepancies resolved through discussion.

Outcome of risk of bias assessment per study was reported as a score on a 10-point scale (one point per item). As cut-off, a risk of bias score of 7/10 or higher was considered low risk, and a score of 6/10 or lower was deemed a high risk of bias by the researchers.

1. Munn Z, Moola S, Lisy K, Riitano D, Tufanaru C. Methodological guidance for systematic reviews of observational epidemiological studies reporting prevalence and cumulative incidence data. *Int J Evid Based Healthc* 2015;**13(3)**:147-53.

## Appendix 6: Meta-analyses of HIV positivity by indicator condition

**Figure 1: Pooled results and estimated proportion tested HIV positive per indicator condition**

**1A: Tuberculosis**


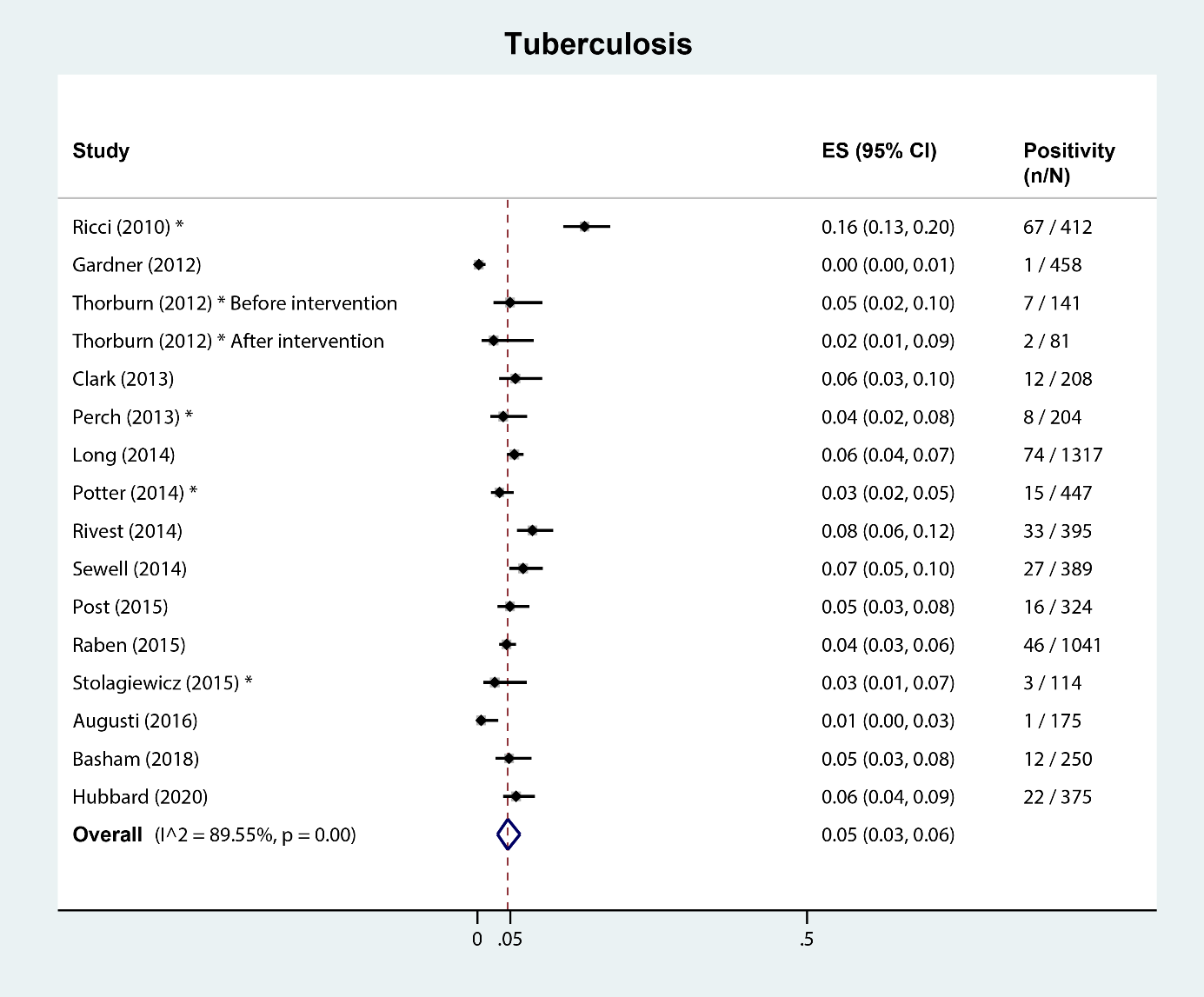


* Other publication types than full-text articles (i.e. abstracts, short communication, and correspondence). ES: estimated proportion. n=cases tested. N=cases identified.

**1B: Hepatitis B and C**


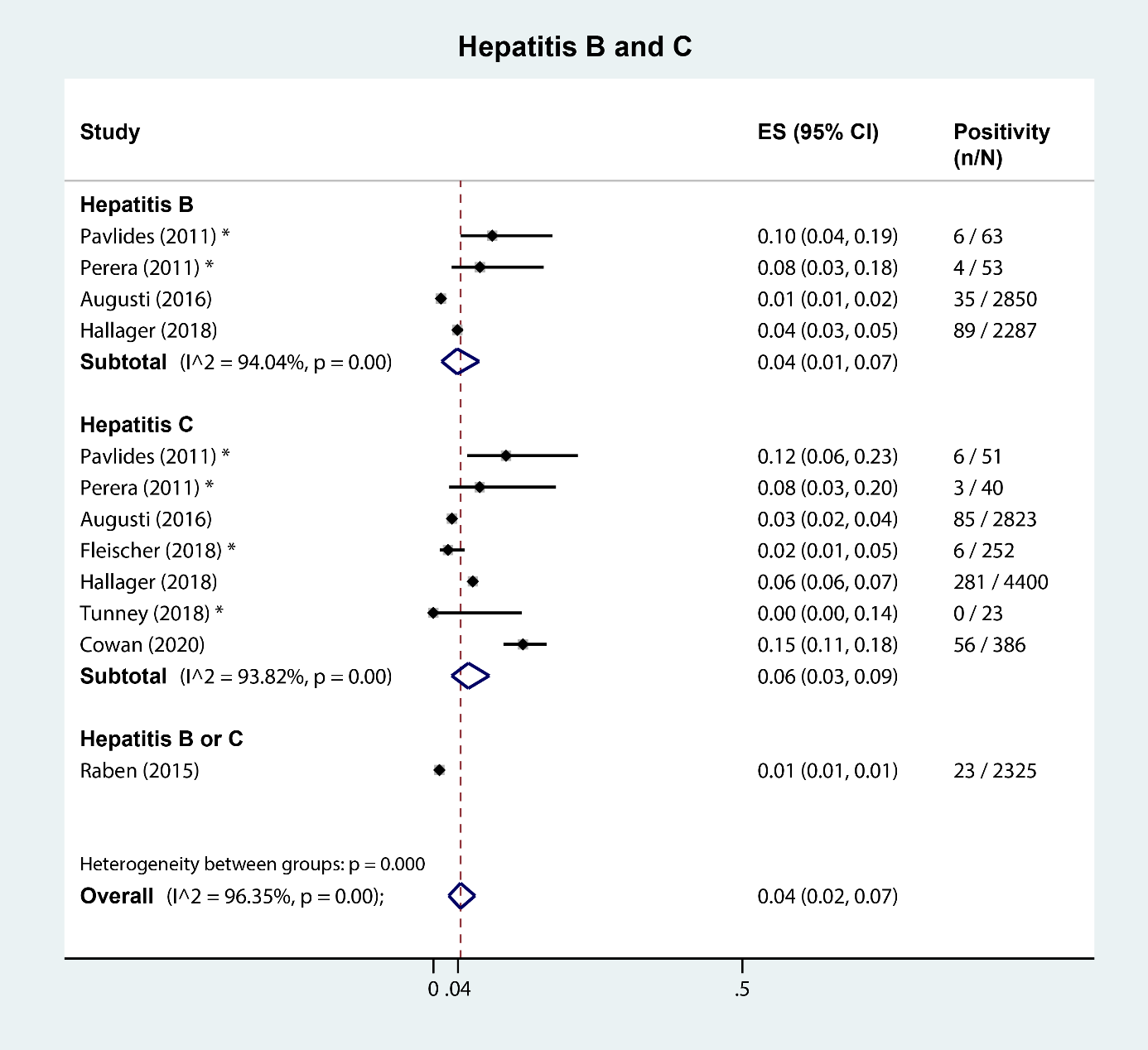


* Other publication types than full-text articles (i.e. abstracts, short communication, and correspondence). ES: estimated proportion. n=cases tested. N=cases identified.

**1C: Malignant lymphoma**


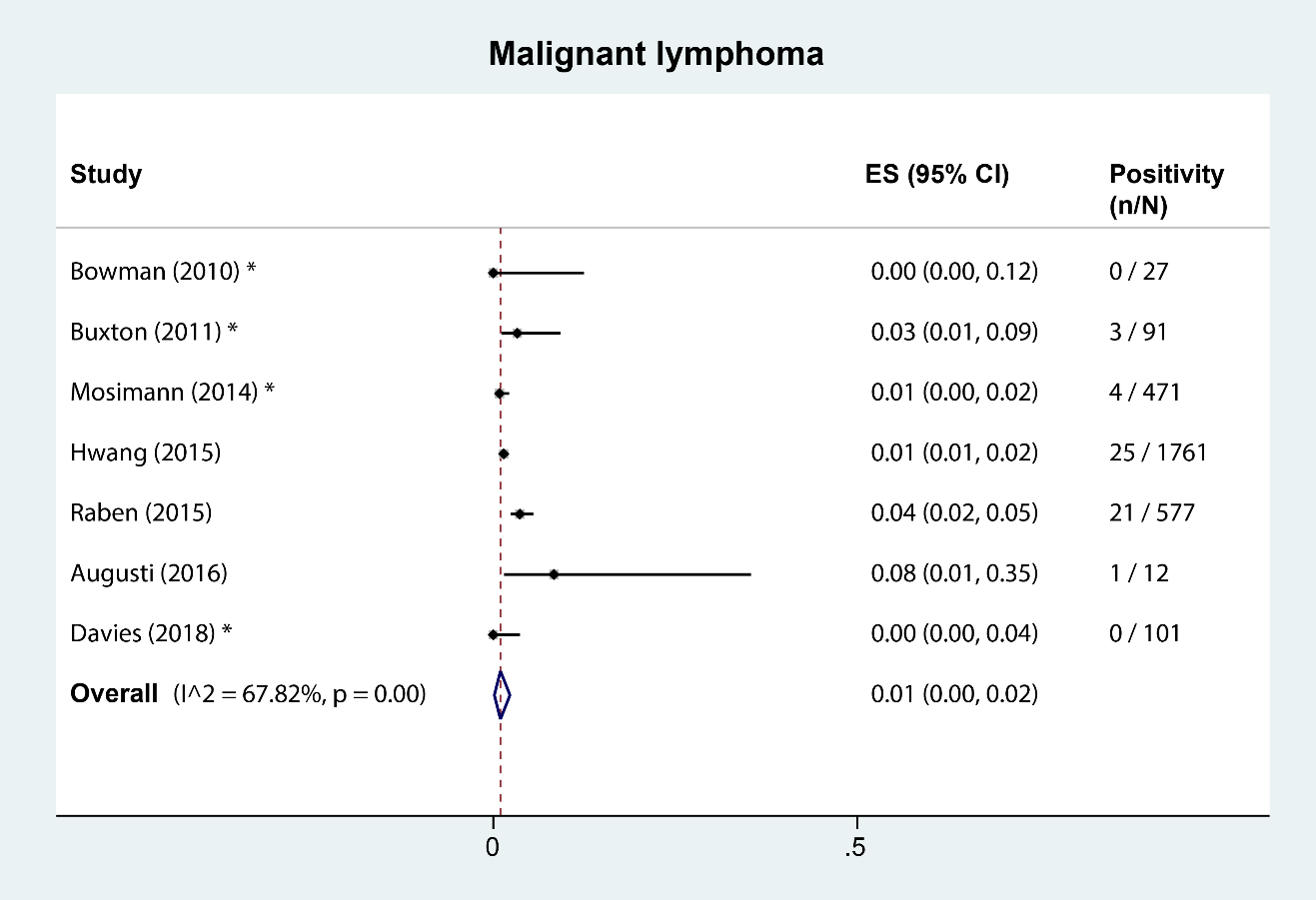


* Other publication types than full-text articles (i.e. abstracts, short communication, and correspondence). ES: estimated proportion. n=cases tested. N=cases identified.

**1D: Cervical carcinoma or cervical intraepithelial neoplasia grade 2+**


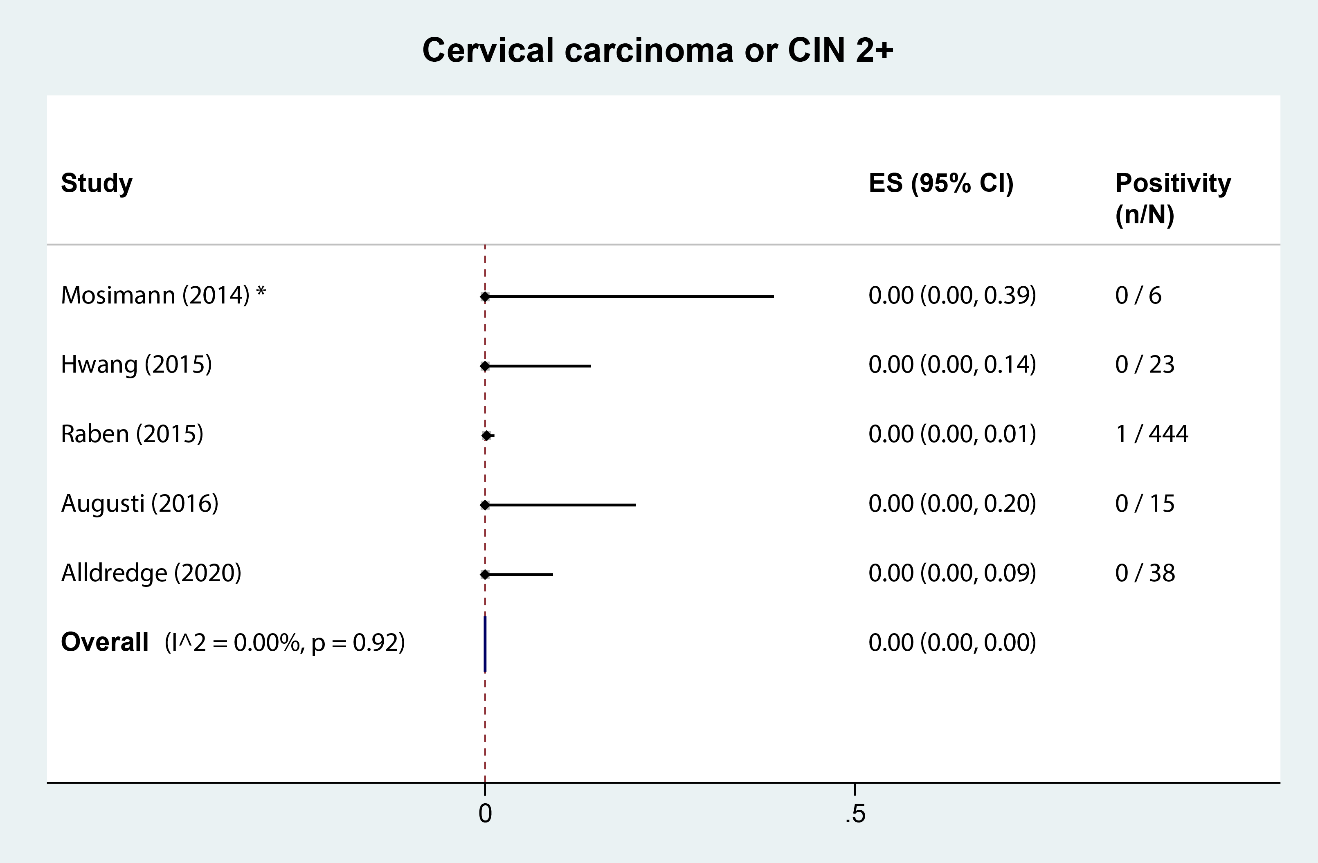


* Other publication types than full-text articles (i.e. abstracts, short communication, and correspondence). CIN: cervical intraepithelial neoplasia. ES: estimated proportion. n=cases tested. N=cases identified.

## Appendix 7: H-TEAM consortium members

**H-TEAM Steering Committee:** J.E.A.M. van Bergen^1;2;3^, G.J. de Bree^4;5^, P. Brokx^6^, U. Davidovich^7;8^, F. Deug^1^, S.E. Geerlings^5^, M. Heidenrijk^4^, E. Hoornenborg^7^, A. Oomen^1^, M. Prins^5;7^, P. Reiss^4;9^, A. van Sighem^9^, M. van der Valk^5^, W. Zuilhof^1^

**H-TEAM Project Management:** N. Schat^4^

**H-TEAM additional collaborators:** M. van Agtmael^10^, J. Ananworanich^11^, D. Van de Beek^12^, G.E.L. van den Berk^13^, D. Bezemer^9^, A. van Bijnen^1^, J.P. Bil^7^, W.L. Blok^12^, S.J. Bogers^5^, M. Bomers^10^, C.A.B. Boucher^14^, A. Boyd^7;9^, W. Brokking^15^, D. Burger^16^, K. Brinkman^13^, N. Brinkman^17^, M. de Bruin^18^, S. Bruisten^7^, L. Coyer^7^, R. van Crevel^19^, C.G. Daans^7;20^; M. Dijkstra^7^, Y.T. van Duijnhoven^7^, A. van Eeden^15^, L. Elsenburg^15^, M.A.M. van den Elshout^7^, E. Ersan^2^, P.E.V. Felipa^7^, T.B.H. Geijtenbeek^21^, J. van Gool^7^, A. Goorhuis^5^, M. Groot^15^, C.A. Hankins^4^, A. Heijnen^22;23^, M.M.J Hillebregt^9^, M. Hommenga^7^, J.W. Hovius^5^, Y. Janssen^17^, K. de Jong^7^, V. Jongen^7^, N.A. Kootstra^24^, R.A. Koup^25^, F.P. Kroon^26^, T.J.W. van de Laar^27;28^, F. Lauw^29^, M.M. van Leeuwen^6^, K. Lettinga^30^, I. Linde^7^, D.S.E. Loomans^7^, I.M. van der Lubben^7^, J.T. van der Meer^5^, T. Mouhebati^1^, B.J. Mulder^7^, J. Mulder^31^, F.J. Nellen^5^, A. Nijsters^1^, H. Nobel^5^, E.L.M. Op de Coul^3^, E. Peters^10^, I.S. Peters^7^, T. van der Poll^5^, O. Ratmann^32^, C. Rokx^33^, M.F. Schim van der Loeff^7;34^, W.E.M. Schoute^13^, J. Schouten^7^, J. Veenstra^30^, A. Verbon^33^, F. Verdult^6^, J. de Vocht^10^, H.J. de Vries^7;34;35^, S. Vrouenraets^30^, M. van Vugt^5^, W.J. Wiersinga^5^, F.W. Wit^5;8^, L.R. Woittiez^5^, S. Zaheri^9^, P. Zantkuijl^1^, M.C. van Zelm^36^, A. Żakowicz^37^, H.M.L. Zimmermann^7^.

1. Soa Aids Nederland, Amsterdam, the Netherlands
2. Department of General Practice, Amsterdam UMC – location AMC, University of Amsterdam, Amsterdam, the Netherlands
3. Epidemiology and Surveillance Unit, Center for Infectious Disease Control, National Institute of Public Health and the Environment, the Netherlands
4. Department of Global Health, Amsterdam UMC – location AMC, and Amsterdam Institute for Global Health and Development, Amsterdam, the Netherlands
5. Department of Internal Medicine, Division of Infectious Diseases, Amsterdam UMC – location AMC, Amsterdam, the Netherlands
6. Dutch Association of PLHIV, Amsterdam, the Netherlands
7. Department of Infectious Diseases, Public Health Service of Amsterdam, Amsterdam, the Netherlands
8. Department of Social Psychology, University of Amsterdam, Amsterdam, the Netherlands
9. Stichting HIV Monitoring, Amsterdam, the Netherlands
10. Department of Internal Medicine, Amsterdam UMC – location VUMC, Amsterdam, the Netherlands
11. US Military HIV Research Program and the Henry M. Jackson Foundation for the Advancement of Military Medicine, Bethesda, United States
12. Center of Infection and Immunity Amsterdam (CINIMA), Department of Neurology, Amsterdam UMC – location AMC, Amsterdam, the Netherlands
13. Department of internal medicine, OLVG – location East, Amsterdam, the Netherlands
14. Department of viro-science, Erasmus Medical Center Rotterdam, Rotterdam, the Netherlands
15. DC Klinieken, Amsterdam, the Netherlands
16. Department of Pharmacy, Radboud University Nijmegen Medical Center, Nijmegen, the Netherlands
17. Primary Care Amsterdam and Almere (Elaa), Amsterdam, the Netherlands
18. Aberdeen Health Psychology Group, Institute of Applied Health Sciences, University of Aberdeen, Aberdeen, United Kingdom
19. Department of Internal Medicine, Radboud University Nijmegen Medical Center, Nijmegen, the Netherlands
20. Center of Expertise on Gender Dysphoria, Amsterdam UMC – location VUMC, Amsterdam, the Netherlands
21. Laboratory of Experimental Immunology, Amsterdam UMC – location AMC Amsterdam, the Netherlands
22. Sexology Center Amsterdam, Amsterdam, the Netherlands
23. GP practice Heijnen & de Meij, Amsterdam, the Netherlands
24. Laboratory for Viral Immune Pathogenesis, Amsterdam UMC – location AMC Amsterdam, the Netherlands
25. Immunology Laboratory, Vaccine Research Center, National Institute of Allergy and Infectious Diseases, National Institutes of Health, Rockville, Maryland, USA
26. Department of Infectious Diseases, Leiden University Medical Center, Leiden, the Netherlands
27. Department of Medical Microbiology, OLVG, Amsterdam, the Netherlands
28. Department of Donor Medicine Research, Laboratory of Blood-borne Infections, Sanquin Research, Amsterdam, the Netherlands
29. Department of Internal Medicine, Medical Center Jan van Goyen, Amsterdam, the Netherlands
30. Department of Internal Medicine, OLVG – location West, Amsterdam, the Netherlands
31. Department of Internal Medicine, Slotervaart Hospital (former), Amsterdam, the Netherlands
32. School of Public Health, Faculty of Medicine, Imperial College London, London, United Kingdom
33. Department of Internal Medicine and Infectious Diseases, Erasmus Medical Center, Rotterdam, the Netherlands
34. Center for Infection and Immunology, Amsterdam (CINIMA), Amsterdam UMC – location AMC, University of Amsterdam, Amsterdam, the Netherlands
35. Department of Dermatology, Amsterdam UMC – location AMC, University of Amsterdam, Amsterdam, the Netherlands
36. Department of Virology, Erasmus Medical Center, Rotterdam, the Netherlands
37. AIDS Healthcare Foundation, Amsterdam, the Netherlands
